# CDK9 inhibition as an effective therapy for small cell lung cancer

**DOI:** 10.1038/s41419-024-06724-4

**Published:** 2024-05-20

**Authors:** L. Valdez Capuccino, T. Kleitke, B. Szokol, L. Svajda, F. Martin, F. Bonechi, M. Krekó, S. Azami, A. Montinaro, Y. Wang, V. Nikolov, L. Kaiser, D. Bonasera, J. Saggau, T. Scholz, A. Schmitt, F. Beleggia, H. C. Reinhardt, J. George, G. Liccardi, H. Walczak, J. Tóvári, J. Brägelmann, J. Montero, M. L. Sos, L. Őrfi, N. Peltzer

**Affiliations:** 1 University of Cologne, Faculty of Medicine and University Hospital Cologne, Department of Translational Genomics, Cologne, Germany; 2 University of Cologne, Faculty of Medicine and University Hospital Cologne, Center for Molecular Medicine Cologne, Cologne, Germany; 3 CECAD Research Center, University of Cologne, Cologne, Germany; 4 Vichem Chemie Research Ltd., Veszprém, Hungary; 5 Department of Experimental Pharmacology, and the National Tumor Biology Laboratory, National Institute of Oncology, Budapest, Hungary; 6 Institute for Bioengineering of Catalonia (IBEC), Barcelona Institute of Science and Technology (BIST), 08028 Barcelona, Spain; 7 Networking Biomedical Research Center in Bioengineering, Biomaterials and Nanomedicine (CIBER-BBN), 28029 Madrid, Spain; 8 Department of Biomedical Sciences, Faculty of Medicine and Health Sciences, University of Barcelona, 08036 Barcelona, Spain; 9 Department of Pharmaceutical Chemistry, Semmelweis University, Budapest, Hungary; 10 Centre for Cell Death, Cancer, and Inflammation (CCCI), UCL Cancer Institute, University College London, London, UK; 11 Cell death, inflammation and immunity laboratory, Institute of Biochemistry I, Centre for Biochemistry, Faculty of Medicine, University of Cologne, Cologne, Germany; 12 Genome instability, inflammation and cell death laboratory, Institute of Biochemistry I, Centre for Biochemistry, Faculty of Medicine, University of Cologne, Cologne, Germany; 13 University Hospital of Cologne, Medical Faculty, Department I for Internal Medicine, Cologne, Germany; 14 University of Cologne, Faculty of Medicine and University Hospital Cologne, Mildred Scheel School of Oncology Cologne, Cologne, Germany; 15 Department of Hematology and Stem Cell Transplantation, University Hospital Essen, University Duisburg-Essen, German Cancer Consortium (DKTK partner site Essen), Essen, Germany; 16 Department of Otorhinolaryngology, Head and Neck Surgery, Faculty of Medicine and University Hospital Cologne, University Hospital of Cologne, Cologne, Germany; 17 Division for Translational Oncology, German Cancer Research Center (DKFZ), The German Consortium for Translational Cancer Research (DKTK), München Partner Site, Ludwig-Maximilian University München, Munich, Germany

**Keywords:** Small-cell lung cancer, Apoptosis

## Abstract

Treatment-naïve small cell lung cancer (SCLC) is typically susceptible to standard-of-care chemotherapy consisting of cisplatin and etoposide recently combined with PD-L1 inhibitors. Yet, in most cases, SCLC patients develop resistance to first-line therapy and alternative therapies are urgently required to overcome this resistance. In this study, we tested the efficacy of dinaciclib, an FDA-orphan drug and inhibitor of the cyclin-dependent kinase (CDK) 9, among other CDKs, in SCLC. Furthermore, we report on a newly developed, highly specific CDK9 inhibitor, VC-1, with tumour-killing activity in SCLC. CDK9 inhibition displayed high killing potential in a panel of mouse and human SCLC cell lines. Mechanistically, CDK9 inhibition led to a reduction in MCL-1 and cFLIP anti-apoptotic proteins and killed cells, almost exclusively, by intrinsic apoptosis. While CDK9 inhibition did not synergise with chemotherapy, it displayed high efficacy in chemotherapy-resistant cells. In vivo, CDK9 inhibition effectively reduced tumour growth and improved survival in both autochthonous and syngeneic SCLC models. Together, this study shows that CDK9 inhibition is a promising therapeutic agent against SCLC and could be applied to chemo-refractory or resistant SCLC.

## Introduction

Lung cancer accounts for 1.76 million deaths worldwide annually, making it the leading cause of cancer-related deaths [1]. Lung cancer is typically divided into two major groups based on histological features: the more common Non-Small Cell Lung Cancer (NSCLC), which comprises 85% of all lung cancers and includes lung adenocarcinomas and squamous cell carcinomas, and Small Cell Lung Cancer (SCLC) [2, 3]. SCLC is a highly aggressive neuroendocrine carcinoma, accounting for 15% of all new lung cancer cases [4, 5]. The disease is characterised by loss of *TP53* and *RB1* [4, 5] as well as early and widespread metastasis, leading to advanced-stage disease at diagnosis in over 85% of patients [6–8]. Standard first-line treatment consists of cisplatin or carboplatin in combination with etoposide and typically elicits initial response rates of over 60%, even in patients with metastases. However, disease recurrence and progression with further resistance to chemotherapy are almost universal [6, 9]. This is reflected in the poor survival rates of only 7% in five years even with the addition of immunotherapy to the first-line treatment, and highlights the need for novel therapies [10, 11].

Kinases are critical components of most oncogenic signalling pathways, which promote tumour growth, migration, invasion, and metastasis and counteract apoptosis induction, thus contributing to resistance to treatment. Cyclin-dependent kinases (CDKs) are proteins with critical roles in cell homoeostasis. They can be divided into two major subgroups: those that modulate the cell cycle and those that regulate transcription. CDK9, a member of the latter subset, is a critical regulator of transcriptional homoeostasis by initiating mRNA transcription elongation [12]. Several studies have also demonstrated that CDK9 interacts with and facilitates target gene activation of the transcription factors NF-κB and c-Myc, among others [12–15]. Owing to these essential regulatory functions, it is no surprise that dysregulation of CDK9 and other CDKs is closely associated with cancer progression [16, 17]. Most tumours rely on continuously activated gene expression for sustained cell cycle progression and anti-apoptotic signals. This has led to the development of CDK inhibitors that may hit several CDKs, including dinaciclib, but also more specific CDK9 inhibitors, such as LDC067, which have emerged as attractive therapeutic options [18–20].

Dinaciclib is a CDK inhibitor, with two active and 11 completed clinical trials to date [21], including breast cancer, pancreatic cancer, melanoma and leukaemia, as well as a phase-III trial for CLL, where it showed improved survival in patients with refractory CLL [22]. Dinaciclib has been tested in a variety of tumour entities, showing efficient killing capacity [23–28]. In NSCLC, dinaciclib treatment alone does not have potent killing activity, although it synergises with TNF-related apoptosis-inducing ligand (TRAIL; also known as Apo2L) treatment [23, 24]. The latter is a cytokine that induces cell death via the extrinsic death receptor apoptosis pathway [29, 30]. Despite being the clinically most advanced CDK9-inhibiting drug, dinaciclib does not only inhibit CDK9 (at an IC_50_ of 4 nM) but also CDK1, CDK2 and CDK5 (with IC_50_ values of 3 nM, 1 nM and 1 nM, respectively) [31]. Efforts to employ more specific inhibitors resulted in the development of NVP-2, a recently derived selective and specific CDK9 inhibitor (at an IC_50_ of 0.5 nM) [32, 33]. Mechanistically, dinaciclib and NVP-2 treatment in cancer cells inhibit CDK9, preventing phosphorylation of RNA pol II and, thus, the initiation of elongation in mRNA transcription. Indeed, it has been shown that CDK9 inhibition decreases the expression of short-lived anti-apoptotic factors cFLIP, cIAP1/2 and MCL-1 in a variety of tumour entities, including NSCLC, pancreatic cancer, colorectal cancer, and melanoma [23, 24]. This, in turn, sensitises the cells to apoptosis. Dinaciclib has also been shown to synergise with the inhibition of Bcl-2 family members in soft tissue sarcoma, ovarian cancer, and SCLC [34–37]. Additionally, the use of dinaciclib in vivo with the addition of immunotherapy abrogated colorectal tumour growth [38].

In this study, we tested the efficacy of CDK9 inhibition in SCLC. We show that dinaciclib displayed high killing potential in mouse and human SCLC but not in NSCLC cell lines. Notably, here we report on a newly developed, highly specific CDK9 inhibitor, VCC972839:01 (VC-1), with comparable anti-tumour activity to dinaciclib. While CDK9 inhibition did not synergise with chemotherapy, it displayed high killing activity on chemotherapy-resistant cells. Mechanistically, CDK9 inhibition led to a reduction of both MCL-1 and cFLIP anti-apoptotic proteins, as first reported by Lemke et al. in NSCLC [24], but causes mainly intrinsic apoptosis. We also observed a weak apoptotic adaptation upon dinaciclib treatment, rendering SCLC cells exquisitely sensitive to this treatment. In vivo, dinaciclib treatment showed potent anti-tumour activity in an autochthonous model of SCLC. Similarly, VC-1 treatment resulted in an anti-tumour response in a subcutaneous SCLC model. Together, this study shows that CDK9 inhibitors represent a promising therapeutic strategy against SCLC and could be applied to chemo-refractory or -resistant SCLC.

## Results

### CDK9 inhibition efficiently kills mouse and human SCLC but not NSCLC cells

**Fig. 1:**
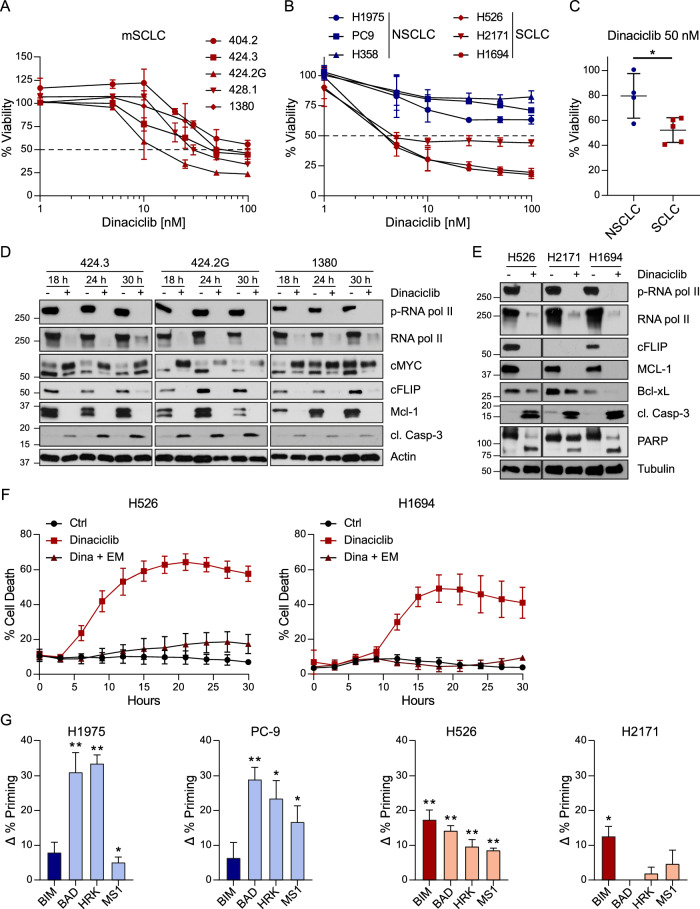
Mouse and human SCLC and NSCLC cells show different sensitivities to dinaciclib. **A** Viability of mouse SCLC cell lines as measured with Cell TiterGlow expressed as % of untreated control (100%) after 30-h treatment with different concentrations of dinaciclib: 1, 5, 10, 20, 25, 30, 50 and 100 nM. Mean + SD, *n* = 3. **B** Viability of human SCLC and NSCLC cell lines after 30-hour treatment 1, 5, 10, 30, 50 and 100 nM of dinaciclib. Mean + SD, *n* = 3. **C** Viability of mouse SCLC compared to NSCLC after 30-h treatment with 50 nM of dinaciclib. Mean + SD, *n* = 3. **D** Mouse SCLC cells were lysed with RIPA buffer after 18, 24 and 30 h treatment with dinaciclib (50 nM) or vehicle. Representative blots of 3 independent experiments. **E** Human SCLC cells were lysed with RIPA buffer after 18 h treatment with dinaciclib (50 nM) or vehicle. Representative blots of 3 independent experiments. p- phospho-, cl. cleaved. **F** Percentage of PI-positive cells after treatment with 50 nM dinaciclib and 5 µM emricasan (EM) as measured by Incucyte. Mean + SD, *n* = 3. **G** Dynamic BH3 profiling after 96 h incubation with 25 nM dinaciclib with the indicated peptides BIM, BAD (BCL-2, BCL-xL, BCL-W dependence), HRK (BCL-xL dependence) and MS1 (MCL-1 dependence). Results expressed as ∆% priming, representing the increase in priming compared to non-treated cells. Values indicate mean ± SEM from at least three independent experiments. Paired t- test of dinaciclib treated vs. ctrl in each condition, **p < 0.01 and *p < 0.05.

To test the effect of dinaciclib on SCLC, we used a panel of human cell lines and mouse cells derived from the autochthonous mouse model of SCLC. This model consists of lung-specific mutations in *Rb1* and *Trp53* (RP) [39]. Dinaciclib treatment induced potent loss of viability and cell death in a dose-dependent manner in SCLC cells, with IC_50_s ranging between 44–124 nM for mouse cell lines and 5–20 nM for human cell lines (Fig. 1A, B and Supplementary Fig. 1a–c). CDK9 inhibition by dinaciclib in murine SCLC cells was confirmed by a decrease in phosphorylated RNA pol II as detected by immunoblotting. Furthermore, we observed downregulation of the anti-apoptotic proteins MCL-1 and cFLIP, as well as cleavage of caspase-3 in the three cell lines assessed at three different time points after treatment (Fig. 1D). Of interest, c-Myc expression was altered, showing an increase in the longer isoform upon treatment with dinaciclib. The same protein expression pattern was observed in human SCLC cells treated with dinaciclib, including downregulation of BCL-xL and increased levels of cleaved Caspase-3 and PARP, indicating activation of apoptosis upon treatment (Fig. 1E). Indeed, cell death induced by dinaciclib was completely blocked by caspase inhibition (Fig. 1F) without causing major changes on cell cycle progression after 24 h of treatment (Supplementary Fig. 1d). Thus, dinaciclib induces caspase-dependent cell death in SCLC cells at nanomolar concentrations.

We then tested whether a more specific CDK9 inhibitor would have a similar killing potential in SCLC. To this end, we used NVP-2, an agent that was described to inhibit CDK9 but not the other CDKs targeted by dinaciclib [32]. Treatment of mouse SCLC cells with NVP-2 kills cells similar to dinaciclib (Supplementary Fig. 1e, f). We also observed inhibition of RNA pol II phosphorylation, which was associated with a decrease in MCL-1 and cFLIP and an increase in cleaved caspase 3, in the cells treated with NVP-2. (Supplementary Fig. 1g).

Downregulation of pro-apoptotic proteins by dinaciclib was also reported in NSCLC. Yet, dinaciclib treatment alone in NSCLC is not sufficient to kill these cells [23, 24]. This prompted us to perform a side-by-side comparison of the cytotoxic capacity of dinaciclib in SCLC *versus* NSCLC. To this end, we used human and mouse cells derived from the *Kras* and *Trp53* (KP) for NSCLC [40]. Dinaciclib had a modest effect on the viability of NSCLC cells as compared to SCLC cells (Fig. 1B, C and Supplementary Fig. 1h). Importantly, the increased sensitivity of SCLC cells to dinaciclib was more evident in the panel of human SCLC as compared to human NSCLC cell lines (Fig. 1C). To further explore the fundamental difference in sensitivities between SCLC and NSCLC to dinaciclib, we performed dynamic BH3 profiling (DBP). DBP measures the overall apoptotic sensitivity or “priming for death” and identifies selective dependencies on anti-apoptotic proteins [41]. We used the BIM peptide to predict treatment cytotoxicity as well as the sensitiser peptides BAD, HRK and MS1 to determine anti-apoptotic protein dependencies in response to dinaciclib. As predicted, SCLC cells are significantly more primed to apoptosis than NSCLC cells in response to dinaciclib as shown by an increased delta priming (Δ% priming) upon treatment with the BIM peptide (Fig. 1G). Regarding the increase in Δ% priming with treatment, we observed that SCLC cells displayed a low degree of Δ% priming, with H2171 cells not being sensitised at all, whereas NSCLC cells showed a much higher degree of Δ% priming upon dinaciclib treatment with all three sensitiser peptides. This suggests anti-apoptotic adaptations to this therapeutic agent (N.B. dinaciclib) in NSCLC (Fig. 1G). Thus, while NSCLC cells show pro-survival adaptation, SCLC cells seem to be less efficient in doing so. Consequently, whereas inhibition of MCL-1, and also slightly BCL-xL, by dinaciclib is sufficient to kill SCLC cells, NSCLC cells are more resistant due to a general lower apoptotic priming and plasticity to rapidly adapt through anti-apoptotic proteins to support survival. Hence, SCLC cells are less able to overcome the killing activity of dinaciclib, which ultimately causes a strong apoptotic response.

### Dinaciclib does not synergise with TRAIL or chemotherapy but potently kills chemotherapy-resistant cells

Because dinaciclib treatment resulted in the downregulation of the anti-apoptotic proteins MCL-1, BCL-xL and cFLIP, we next tested whether it could synergise with therapies that target either the intrinsic or extrinsic cell death pathways. The combination of CDK9 inhibition and TRAIL treatment was reported to be highly efficient in various tumour entities, including NSCLC and pancreatic cancer [23, 24, 42]. Hence, we speculated that the combination of dinaciclib and TRAIL treatment would also be highly efficacious in this tumour entity. However, in contrast to NSCLC cells, combined dinaciclib and TRAIL treatment failed to show a synergistic or additive effect in SCLC cells (Supplementary Fig. 2a). In line with this observation, the combination of TRAIL and dinaciclib treatment failed to induce caspase-8 cleavage in H1694 SCLC cells, whereas it successfully did so in the NSCLC cell lines tested. Remarkably, expression of caspase-8 in H2171 SCLC cells is much lower than in NSCLC (Supplementary Fig. 2b), in line with previous reports showing that lack or reduced expression of caspase-8 is frequent in SCLC [10, 43, 44].

**Fig. 2:**
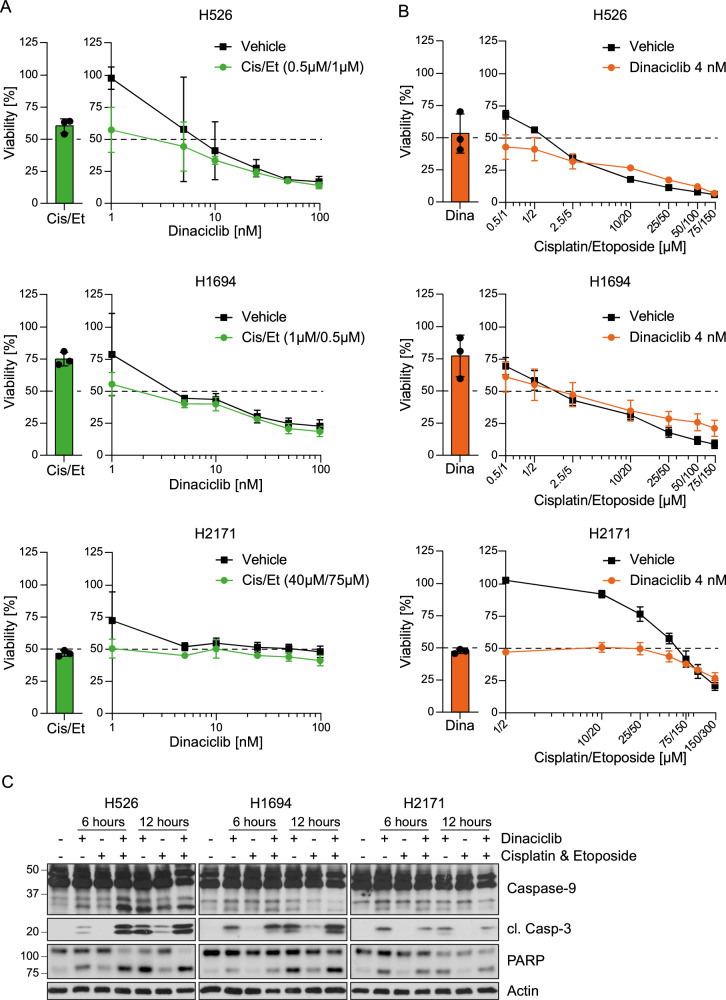
CDK9 inhibition shows no synergy with standard chemotherapy in SCLC. **A** Human cell lines were treated with increasing doses of dinaciclib (1, 5, 10, 25, 50 and 100 nM) for 30 h in the presence or absence of a combination of cisplatin and etoposide. H526: Cis 0.5 µM, Et 1 µM. H1694: Cis 1 µM, Et 0.5 µM. H2171: Cis 40 µM, Et 75 µM. Mean + SD, *n* = 3. Viability was measured by CTG and expressed as a percentage of the viability of control. **B** Human cell lines were treated with increasing doses of cisplatin and etoposide for 30 h in the presence or absence of 4 nM dinaciclib. Mean + SD, *n* = 3. **C** SCLC cells were lysed with RIPA buffer after 6 and 12 h of treatment with either 25 nM dinaciclib and/or cisplatin and etoposide. H526 & H1694: Cis 20 µM, Et 50 µM. H2171: Cis 40 µM, Et 100 µM. Representative blots of 3 independent experiments. Cis cisplatin, Et etoposide, cl. cleaved.

To test whether dinaciclib could synergise with standard-of-care chemotherapy, we treated SCLC cells with dinaciclib and a combination of cisplatin and etoposide. We co-treated the cells with a fixed concentration of cisplatin and etoposide while increasing the dose of dinaciclib, and vice versa. While dinaciclib succeeded in decreasing cell viability in a dose-dependent manner, we observed no synergistic or additive effect when combined with cisplatin and etoposide within the tested concentration range (Fig. 2A, B, Supplementary Fig. 3a, b). Among the human cell lines evaluated, H2171 showed a markedly increased tolerance to cisplatin and etoposide as higher doses of the chemotherapeutic agents are needed to reach the same reduction in viability when compared to the other cell lines tested (Fig. 2B). Yet, it responded as well to dinaciclib as the other SCLC cell lines (Fig. 1B). We then examined the kinetics of caspase cascade activation downstream of mitochondrial damage upon dinaciclib alone or in combination with chemotherapy (cisplatin and etoposide). We observed that both caspase-9 and caspase-3 are readily activated after 6 h of dinaciclib treatment, whereas they are poorly activated by chemotherapy alone. Curiously, combined dinaciclib and chemotherapy treatment enhanced the activation of these caspases at an early time point (Fig. 2C). However, at later time points, the difference in caspase activation in cells treated with dinaciclib or the triple combination is no longer observed (Fig. 2C). Although, at 6 h the triple combination showed higher caspase activity than the single agents, we could not observe faster kinetics of cell death by dinaciclib and chemotherapy as compared to dinaciclib alone (Supplementary Fig. 3C). This correlates with the fact that we observed comparable levels of PARP cleavage between cells treated with either dinaciclib or the triple combination at early and late time points (Fig. 2C). Next, we studied the potential additive effect of NVP-2 and chemotherapy; in line with our findings with dinaciclib, there was no further decrease in viability upon the combination treatment (Supplementary Fig. 3d).

**Fig. 3:**
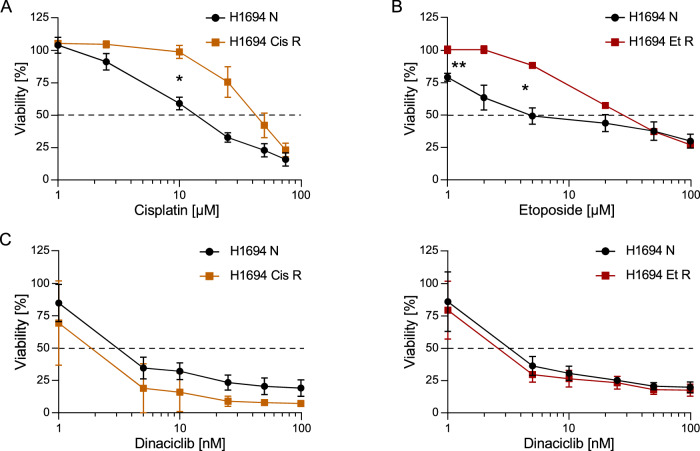
Dinaciclib treatment efficiently kills SCLC cells with acquired resistance to chemotherapy. **A** SCLC cells with acquired resistance to cisplatin (Cis R) and the naïve parental cell line (N) were treated with increasing doses of cisplatin (0.5, 1, 2.5,10, 25, 50 and 75 µM) for 30 h. Mean + SD, *n* = 3. **B** SCLC cells with acquired resistance to etoposide (Et R) and the naïve parental cell line (N) were treated with increasing doses of etoposide (1, 2, 5, 20, 50 and 100 µM) for 30 h. **C** Resistant and naïve cell lines were treated for 30 h with increasing doses of dinaciclib (1, 5, 10, 25, 50 and 100). Mean + SD, *n* = 3. Viability was measured by CTG and expressed as a percentage of the viability of control. Two-way ANOVA with Geisser-Greenhouse correction. *p-adj<0.05, **p-adj < 0.01.

Given the high killing potential of dinaciclib alone, we examined its activity in cells rendered resistant to chemotherapy by chronic exposure. H1694 cells were cultured with increasing doses of cisplatin or etoposide over time, reaching tolerance to 4 and 3 µM of the drug, respectively. We first confirmed the acquired resistance by titrating the chemotherapy agents, which showed an increase in viability compared to the naïve cell line when treated with the corresponding drugs (Fig. 3A, B). Next, we tested the sensitivity of these cells to dinaciclib. We found that the resistant cells had a comparable sensitivity to dinaciclib than the naïve parental cell line (Fig. 3C). Notably, there was no synergism between dinaciclib and cisplatin (Supplementary Fig. 4a, b) or etoposide (Supplementary Fig. 4c, d) in the corresponding resistant cells.

Taken together, this data indicates that although dinaciclib treatment does not synergise with chemotherapy, it successfully kills chemo-resistant cells, possibly offering an alternative therapy in these cases.

### Development of a novel specific CDK9 inhibitor

**Fig. 4:**
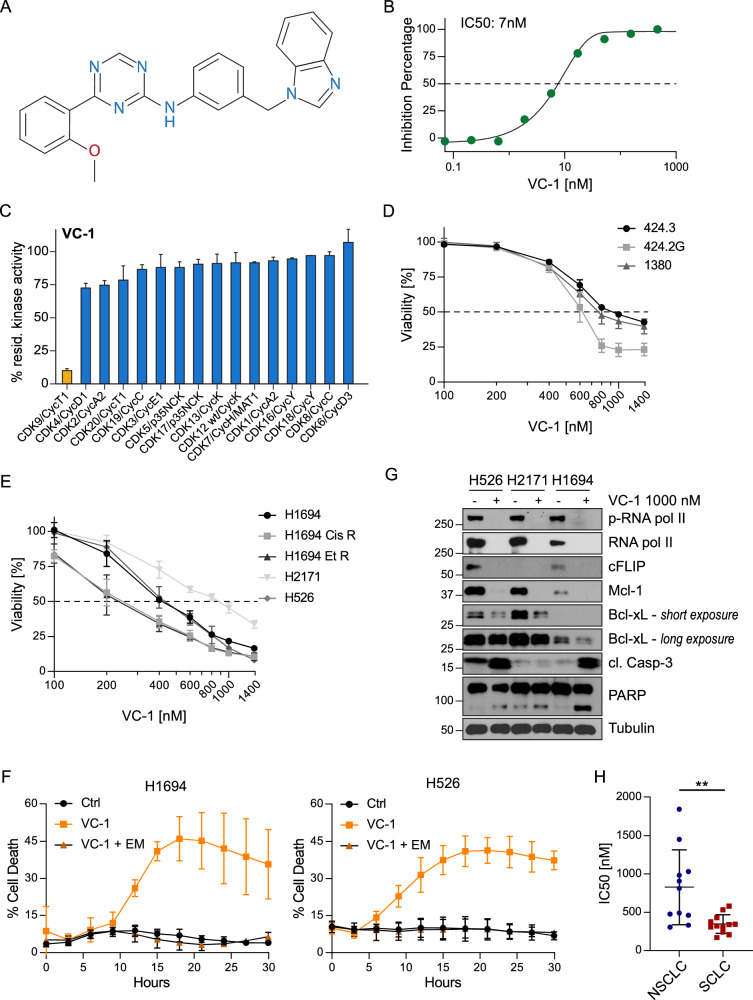
A new class of CDK9 inhibitor. **A** Chemical structure of the compound VC-1. **B** Percentage of inhibition of CDK9 activity calculated from a fluorescence polarisation shift assay. **C** Radiometric selectivity profile assay measuring kinase activity of 16 CDK/Cyclin pairs. Mean + SD, *n* = 2. **D**, **E** Viability was measured by CTG and expressed as a percentage of the viability of Control after 30-hour treatment with different concentrations of VC-1 (100, 200, 400, 600, 800, 1000 and 1400 nM) Mean + SD, *n* = 3. **F** Percentage of PI-positive cells after treatment with 1 000 nM VC-1 and 5 µM emricasan (EM) as measured by Incucyte. Mean + SD, *n* = 3. **G** Human SCLC cells were lysed with RIPA buffer after 18 h of treatment with 1000 nM VC-1 or vehicle. Representative blots of 2 independent experiments. **H** IC_50_ [μM] of twelve SCLC and eleven NSCLC human cell lines after 72 h of treatment with VC-1. Unpaired t-test NSCLC vs. SCLC **p = 0.0034.

To understand if CDK9 inhibition is responsible for the killing capacity of dinaciclib, we used a novel inhibitor, VCC972839:01 (VC-1), that specifically inhibits CDK9 but not other CDKs (Fig. 4A). This molecule was designed to target the hinge region of the kinase, which is responsible for binding to ATP. When modelling the interactions of VC-1 and the crystal structure of CDK9 (PBD ID: 4BCF) by docking, we observe two H-bonds between the small molecule and the Cys106 in the hinge region of the CDK (Supplementary Fig. 5a, b). The triazine N of VC-1 is a H-acceptor and interacts with the peptide NH of Cys106, while the amino NH of VC-1 is a H-donor and interacts with the peptide carbonyl oxygen of Cys106, thus establishing a two-way interaction with the same amino acid within the ATP binding site. We also observe an interaction between the indole ring of the inhibitor and the Phe30 by aromatic stacking (Supplementary Fig. 5a, b). When comparing with the docking model of dinaciclib in the ATP binding site, we observe the same H-bond interactions with Cys106 and an additional H-bond interaction with two possible amino acids, either Asn154 or Asp167 (Supplementary Fig. 5c).

We determined the affinity of VC-1 to CDK9 by a competitive assay measuring fluorescence polarisation, which showed a binding IC_50_ of 7 nM (Fig. 4B). We then tested the specificity of the inhibitor to CDK9 through a radiometric selectivity profile assay, which showed that only the CDK9/CyclinT1 pair was inhibited from a panel of 16 CDK/Cyclin pairs (Fig. 4C).

Next, we assessed the cytotoxicity of VC-1 in mouse and human SCLC. In line with our findings using dinaciclib, VC-1 decreased the viability in a dose-dependent manner in normal SCLC cells, as well as in the chemotherapy resistant cell lines (Fig. 4D, E). Moreover, the VC-1 induced cell death was blocked by caspase inhibition (Fig. 4F), without impacting cell cycle progression (not shown). Protein analysis by immunoblotting showed that VC-1 treatment induces a robust decrease in total and phosphorylated RNA pol II, as well as cFLIP, MCL-1 and BCL-xL (Fig. 4G). Furthermore, we observed increased cleavage of caspase-3 and PARP in all cell lines except for H2171, which showed only a slight increase of the active caspase and cleaved PARP. This aligns with the results from Fig. 4E, where H2171 maintained a higher viability than the other cell lines at the concentration tested (1 µM of VC-1).

Of note, SCLC cell lines showed more sensitivity to treatment with VC-1 than NSCLC cells, as reflected by their lower IC_50_s (Fig. 4H, Supplementary Fig. 5d), similar to what we observed with dinaciclib treatment. These results show that VC-1 efficiently and specifically inhibits CDK9 and induces apoptosis in SCLC cells.

### CDK9 inhibition significantly reduces tumour growth and prolongs survival

**Fig. 5:**
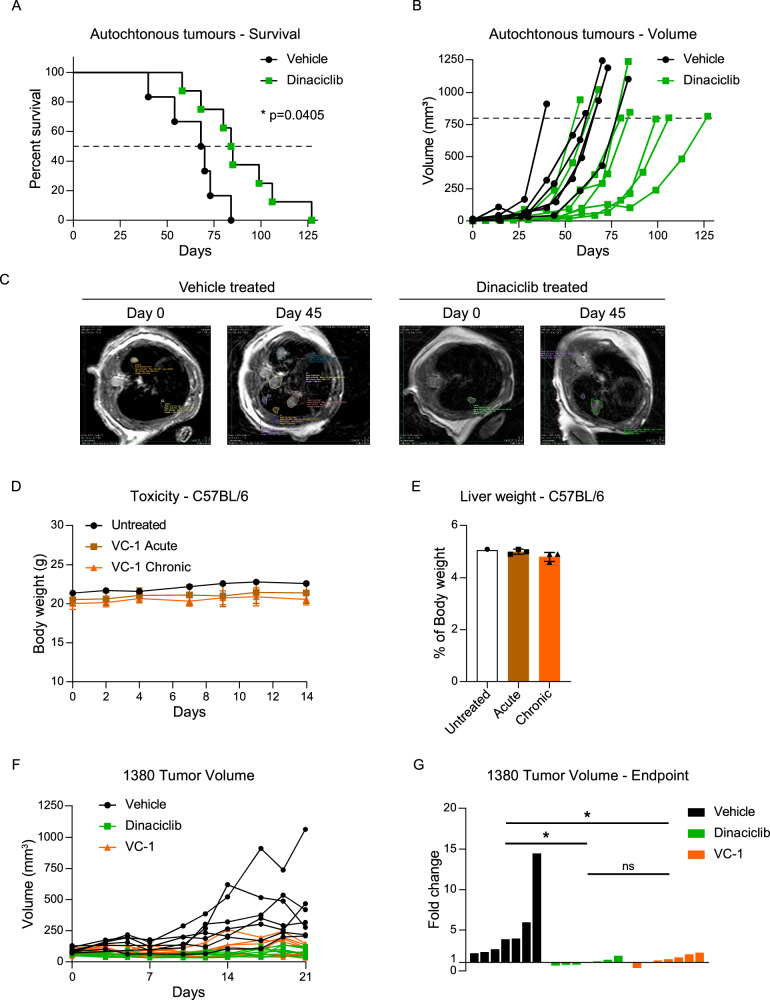
CDK9 inhibition significantly reduces tumour growth and prolongs survival. **A** Survival curve for mice bearing tumours treated with dinaciclib. Log-rank (Mantel–Cox) test. *p = 0.0405. Treatment began upon tumour establishment with either dinaciclib (30 mg/kg) or vehicle (10% Hydroxypropyl Beta Cyclodextrin) twice per week, followed by a week of drug holiday until endpoint criteria were met (tumour volume >800 mm^3^). Tumours were measured every 14 days. Vehicle treated *n* = 6, dinaciclib treated *n* = 8. **B** Volume of autochthonous SCLC tumours as measured by MRI and the Horos software. **C** Representative images of MRI scans of vehicle and dinaciclib-treated mice since tumour volume reached >1 mm^3^ and 45 days after. **D** Weight of C57BL/6 mice treated with a single i.p. injection of 40 mg/kg of VC-1 (Acute) or three injections per week of 20 mg/kg (Chronic). **E** Percentage of liver weight with respect to the body weight of each mouse on day 14 of **D**. **F** 1380 cells were injected subcutaneously on the flank of C57BL/6 mice. Treatment consisted of either VC-1 (20 mg/kg), dinaciclib (30 mg/kg) or vehicle (10% Hydroxypropyl Beta Cyclodextrin) three times per week (VC-1) or twice per week, followed by a week of drug holiday (dinaciclib). Tumours were measured three times per week, *n* = 7 for each group. **G** Individual tumour volumes of **D** at day 21 expressed as fold change from day 0. One-way ANOVA, Dunnet’s multiple comparison test. *p-adj. < 0.05.

We next proceeded to test the efficacy of CDK9 inhibition in vivo using a syngeneic subcutaneous model. We used dinaciclib, which has been tested in many clinical trials and is closer to the clinic than any other CDK inhibitor. Since we observed comparable efficacy of CDK9 inhibition with and without chemotherapy, we tested dinaciclib as a potential single treatment. Accordingly, we injected mouse-derived SCLC cells subcutaneously and treated mice once we observed palpable tumours until endpoint size was achieved. We tried two different cell lines: 1380 and 424.3, and found that in both cases, dinaciclib treatment delayed tumour growth (Supplementary Fig. 6a, c) and improved survival (Supplementary Fig. 6b, d) compared to vehicle controls. Of note, mice injected with cell line 424.3 presented ulcerations and the experiment was terminated prematurely (Supplementary Fig. 6a, b). We did not observe any significant change in body weight between treated and control mice injected with either cell line (Supplementary Fig. 6e, f). To provide further preclinical rationale for the efficacy of dinaciclib treatment, we tested its anti-tumour properties in the well-established autochthonous RP-SCLC model. Briefly, RP mice (*Rb1*^*flox/flox*^; *Trp53*^*flox/flox*^) were inhaled with non-replicative Cre expressing Adenovirus to induce the deletion of *Rb1* and *Trp53*. This co-deletion in the lung has been shown to induce SCLC in mice. Excitingly, dinaciclib treatment significantly improved survival and reduced tumour burden as assessed by MRI-scan (Fig. 5A–C).

Next, we explored the potential of the VC-1 inhibitor as an anti-cancer drug in vivo. We first assessed the toxicity of chronic or acute application of the drug. The results show that VC-1 has no adverse effect in mice, as it does not significantly affect the liver or body weight (Fig. 5D, E). Having confirmed the safety of VC-1, we tested its anti-tumour efficacy in a syngeneic subcutaneous model. Remarkably, three weeks of VC-1 treatment resulted in a significant reduction in tumour growth, which was comparable to the anti-tumoural effect of dinaciclib (Fig. 5F, G). There were no differences in body weight among the different experimental groups across the experiment (Supplementary Fig. 6g). Hence, our results suggest that CDK9 inhibition by either dinaciclib or VC-1 treatment could impair SCLC tumour growth and that both drugs, especially VC-1, are well tolerated in vivo.

## Discussion

In this study, we address the need for novel therapies for the treatment of SCLC by presenting dinaciclib and VC-1 as promising drug candidates. Our results align with multiple reports stating that the killing capacity of dinaciclib is mainly due to the decrease in the levels of anti-apoptotic proteins in malignant cells [34, 35, 45, 46]. Furthermore, we show that CDK9 inhibition kills mainly by intrinsic apoptosis and has no major effect on cell cycle progression. Intriguingly, we observed that SCLC cells are more primed to cell death by CDK9 inhibition, compared to NSCLC cells. However, SCLC cells present diminished apoptosis adaptation in response to dinaciclib as compared to NSCLC, potentially explaining the exquisite sensitivity of SCLC to this therapy.

The first-line treatment for SCLC has seen few changes over the past 30 years, except for the approval of the PD-L1 inhibitor atezolizumab in 2018 by the FDA. Clinical trials with atezolizumab, in combination with etoposide and carboplatin, led to an increase in overall survival of 2 months [47, 48]. We hypothesise that SCLC patients could benefit further from this multi-component treatment when combining it with dinaciclib since it has been reported that dinaciclib treatment elicits the expression of type I IFN response genes and release of DAMPs from tumour cells [38, 49]. This contributes significantly to immune recruitment and recognition. Indeed, the combined dinaciclib/PD-1 therapy in triple-negative breast and colorectal cancers in vivo is associated with elevated infiltration and activation of CD8+ T and dendritic cells [38, 49]. Hence, it would be interesting in the future to test whether dinaciclib or CDK9 inhibition would be able to synergise with immunotherapy in SCLC.

A combination of dinaciclib with specific inhibitors has been proposed before. In SCLC cells, co-treatment of dinaciclib with the BCL-2, BCL-xL, and BCL-w inhibitor Navitoclax was shown to be synergistic [34]. The combinatory effect of the decrease in the levels of MCL-1 by dinaciclib plus the inhibition of the remaining members of the BCL-2 family by navitoclax proves particularly effective in SCLC, as it has been shown that SCLC cell lines display addiction to BCL-2, BCL-xL or MCL-1 for survival [35]. Yet, we could not observe synergism with drugs that induce DNA damage (e.g., cisplatin, etoposide) and activate apoptosis through BCL-2 family members, possibly because the loss of P53 in SCLC alters the cellular response to DNA damage. This lack of synergy can also be explained by the fact that both therapies activate exclusively mitochondrial apoptosis in SCLC, with strong early caspase-9 and -3 cleavage, and no caspase-8 activation. The high apoptosis priming observed in SCLC cells upon dinaciclib treatment alone may also contribute to the lack of synergy with chemotherapy, as both treatments fully engage apoptosis in these cells. Hence, it is conceivable that dinaciclib treatment saturates the apoptotic response and that, based on inefficient apoptotic adaptation in some SCLC cell lines, there is no further sensitisation upon treatment with agents that target mitochondrial cell death. This conclusion does not go in line with studies that showed a synergistic effect between dinaciclib and BH3-mimetics discussed above. This could be due to the use of different SCLC cell lines. Indeed, not all SCLC cell lines tested here respond equally to dinaciclib or show similar apoptotic adaptation. Although our results show lower overall adaptation in SCLC cells as compared to NSCLC, H526 SCLC cells are slightly, albeit significantly, sensitive to BH3 peptides, whereas H2171 cells are not. This reinforces the need for personalised medicine and the importance of profiling tumour-derived cells for treatment stratification. Of even higher importance, in this study we show that dinaciclib not only efficiently kills naïve SCLC cells but also cells with intrinsic and acquired resistance to chemotherapy. This finding is particularly important because resistance to chemotherapy upon relapse remains one of the major challenges when it comes to treating SCLC.

An interesting observation derived from protein analysis, is that the long isoform of c-Myc was increased after treatment with dinaciclib. We speculate that dinaciclib may shift the expression of c-Myc to the isoform which was reported to inhibit proliferation. This is interesting in the context of SCLC since c-Myc is known to guide tumour evolution between different subtypes of SCLC [50, 51]. Therefore, even though dinaciclib did not synergise with cisplatin and etoposide, chemotherapy treatment could still benefit from the addition of dinaciclib by blocking the plasticity between subtypes and preventing the emergence of resistance [52]. The effect of dinaciclib on SCLC cellular plasticity and subtypes warrants further investigation.

In this study, we also introduce VC-1, a novel and specific CDK9 inhibitor. VC-1 showed inhibition of SCLC cell viability with IC50s in the nanomolar range. With an IC_50_ to CDK9 of 7 nM (Fig. 4A), it provides an effective and alternative tool to NVP-2 to study CDK9 inhibition. Furthermore, its anti-cancer activity in vivo positions it as an exciting candidate for the treatment of SCLC along with dinaciclib. We anticipate that the use of CDK9-specific inhibitors such as VC-1 would elicit less untoward adverse effects caused by blocking other CDKs, such as CDK1, 2 and 5, as is the case with dinaciclib. However, it is important to acknowledge that the use of VC-1 would not necessarily solve the issue of development of resistance since the mechanism of CDK9 inhibition between these two compounds is equivalent.

In summary, our work sets the basis for the use of dinaciclib in chemotherapy-resistant SCLC and a rationale to introduce specific CDK9 inhibitors with comparable anti-tumour efficacy and safety profile. Considering the minor progress made in SCLC treatment over the last 30 years, CDK9 inhibition could bring us closer to improving the outcome of treatments against this disease. Further studies combining CDK9 inhibition with immune checkpoint blockade in SCLC might show improved anti-tumour immunity, thus improving the outcome of immunotherapies in SCLC patients.

## Materials and methods

### Cell lines

The mouse NSCLC cell lines KP-1, KP-2, KP-3 and KP-5 were kindly provided by A. Montinaro, the mouse SCLC cell lines 404.2, 424.3, 424.2G, and 428.1, were derived directly from lung tumours of the RP mouse model for SCLC, driven by loss of *Trp53* and *Rb1*. The 1380 cell line was derived from the same model but was additionally injected in the lungs of a recipient mouse C57BL/6J mouse and re-isolated after successful in vivo growth. All cell lines but 1380 were cultured in DMEM (Gibco | Thermo Fisher Scientific, Billings, MT, USA, cat# 10566016) supplemented with 10% Fetal Bovine Serum (FBS) (Gibco, cat# 10270106). 1380 was cultured in RPMI 1640 (Gibco, cat# 21875034) supplemented with 10% FBS. H526, H2171, H1975, PC-9, H358 and H1694 cells were cultured as stated by their suppliers. All media were supplemented with the antibiotic Primocin (Invivogen, San Diego, CA, USA, cat# ant-pm-1).

### Drugs and antibodies

The following inhibitors and drugs were used at the indicated concentration unless otherwise specified in the Figure or Figure legends: dinaciclib 50 nM (Selleck-Chemicals, Houston, TX, USA, cat# S2768), NVP-2 50 nM (Selleck-Chemicals Cat# S8981), etoposide (Absource diagnostic, Munich, Germany, cat# S1225-0100), cisplatin (Selleck-Chemicals, cat# S1166), Propidium Iodide (Sigma-Aldrich, Cat# P4864). Recombinant iz-huTRAIL was provided by H. Walczak. Antibodies against the following antigens were used: RNA pol II RBP1 pSer2 (Biolegend, San Diego, CA, USA, cat# 920204), RNA pol II (Biolegend, cat# 904001), MCL-1 (Cell Signalling, Danvers, MA, USA, cat# 5453), cFLIP (Cell Signalling, cat# 56343), C-Myc (Cell Signalling, cat# 5605), PARP (BD, Franklin Lakes, NJ, USA, cat# 556362), caspase 9 (Abcam, Cambridge, UK, cat# 202068), caspase 8 (Cell Signalling, cat# 9746), cleaved caspase 3 (Cell Signalling, cat# 9664), β-Actin (Sigma-Aldrich, cat# A1978), Tubulin (Sigma-Aldrich, cat# T9026), Rabbit IgG (SouthernBiotech, Birmingham, AL, USA, cat# 4050-05), Mouse IgG (SouthernBiotech, cat# 1031-05). VC-1 was produced and provided by Vichem Chemie Research Ltd.

### Cell viability and cell death assays

Adherent cells were seeded the day before the experiment at 6000 cells per well of a black 96-well plate. The following day the cells were treated with the specified drugs for 30 h. For suspension cells, 10,000 cells per well were seeded and treated simultaneously in a 96-well plate with the specified drugs for 30 h. Cell viability was assessed using the CellTiter-Glo assay (Promega, Madison, WI, USA, Cat# G7571) according to the manufacturer’s instructions. Luminescence was determined using a Tecan infinite M-plex (Tecan, Männedorf, Switzerland). For IC_50_ determination of VC-1, the compound was pre-printed using the D300e Digital Dispenser (Tecan). Viability was determined after 72 h by CellTiter-Glo. The cell lines tested were: NSCLC: H2172, H23, HCC4006, A549, H1568, H1299, H2110, HCC15, PC9, HCC827, H522; SCLC: H196, H1048, GLC2, H82, H1092, GLC1, H526, H524, GLC8, H69, H841, H2029.

The IncuCyte™ Live-Cell Imaging system was used for monitoring cytotoxicity as determined by PI uptake. A 96-well plate was seeded with 2 × 10^4^ cells per well with the treatments specified in each figure. Pictures were taken every 3 h, and the percentage of dead cells was determined by quantifying PI-positive cells using the Cell-by-cell function of the IncuCyte analysis software.

### Dynamic BH3 profiling

Dynamic BH3 profiling was performed as previously described [41]. Briefly, 3 × 10^5^ cells were incubated with targeted therapies (or DMSO in the control condition) for 16 h at 37 °C. Afterwards, cells were stained with the viability marker Zombie Violet (423113, BioLegend, Koblenz, Germany) for 10 min at room temperature (R.T.) and then washed with PBS and resuspended in 330 µl of MEB (150 mM mannitol, 10 mM hepes-KOH pH 7.5, 150 mM KCl, 1 mM EGTA, 1 mM EDTA, 0.1% BSA, 5 mM succinate). Simultaneously, 12 different peptide solutions were prepared in MEB with 0.002% digitonin (D141, Sigma-Aldrich). The final concentration of each peptide solution was: 10, 3, 1, 0.3, 0.1, 0.03, and 0.01 µM of BIM BH3 peptide, 10 µM of BAD BH3 peptide, 100 µM of HRK BH3 peptide, 10 µM of MS1 BH3 peptide, 25 µM of alamethicin (BML-A 150-0005, Enzo Life Sciences, Lörrach, Germany) and DMSO in the control condition. Subsequently, 25 µl of cell suspensions were incubated with 25 µl of each peptide solution in a 96-well plate (3795, Corning, Madrid, Spain) for 1 h at R.T., followed by fixation with formaldehyde and further staining with cytochrome c antibody (Alexa Fluor® 647—6H2.B4, 612310, BioLegend). Individual DBP analyses were performed 16 hs after dinaciclib treatment (25 nM) in triplicates for DMSO, alamethicin, multiple BIM BH3 concentrations, BAD, HRK, and MS1 BH3 peptides. The different analyses were performed with a high-throughput flow cytometry Cytek® Aurora Spectral Flow cytometer (Cytek Bioscience, Freemont, CA, USA). % priming represents the maximum % cytochrome c released obtained after BH3 peptide exposure and Δ% priming stands for the maximum difference between treated cells vs non-treated cells.

### Cell cycle analysis

1.25 × 10^6^ (H526, H1694 and H2171) or 3 × 10^5^ (H526 and H1975) cells were treated with either dinaciclib (50 nM). After 24 h cells were collected, washed with PBS, fixed with ethanol 70% and stained with PI. Cell cycle distribution was assessed by DNA content detected by flow cytometry (BD FACSymphony A3 Flow Cytometer, BD Biosciences, Germany).

### Western blot analysis

Cells were treated as indicated and then lysed in RIPA lysis buffer with 1× cOmplete Protease-inhibitor cocktail (Sigma-Aldrich, cat# 11697498001) and 1× PhosSTOP (Roche, Basel, Switzerland, cat# 4906837001)). Proteins were separated using 4–15% Mini- or Midi-PROTEAN®-TGXTM-gels (Bio-Rad, Hercules, CA, USA, Cat# 4561086, Cat# 5671085) with Tris/glycine/SDS running buffer. Proteins were transferred on Mini- or Midi- 0.2 µm nitrocellulose membranes (Bio-Rad, Cat# 1704158, Cat# 1704159) using the Trans-Blot® Turbo™ Transfer System (Bio-Rad). Proteins were detected with antibodies as indicated.

### In vivo toxicity assessment of VC-1

To evaluate the safety of VC-1, acute and chronic toxicity was assessed. A single injection of 40 mg/kg (Acute, *n* = 3), or three injections per week for two weeks (Chronic, *n* = 3) were administered to the animals intraperitoneally (i.p). Control animals (*n* = 1) were injected with vehicle. Animals were monitored three times per week for general condition (body weight, behaviour, etc). On day 14, mice were sacrificed by cervical dislocation and liver weight was determined.

### In vivo tumour studies

Adult C57BL/6 mice (6–8 weeks) were housed in individually ventilated cages (IVC) systems and given food and water ad libitum. The temperature of the animal facility was 23 °C with a 12-h light/dark cycle. Two models of SCLC were used and are described below:

#### Autochthonous mouse model

Mice harbouring the genetic modifications in the genes *Rb1*^flox/flox^ *Trp53*^flox/flox^ (RP model) [39] were used to induce SCLC. For induction of lung tumours, 8–12-weeks-old mice were anesthetised with Ketamin (100 mg/kg) and Xylazin (10 mg/kg) by intraperitoneal injection followed by intratracheal inhalation of replication-deficient adenovirus expressing Cre (Ad5-CMV-Cre, 2.5 × 10^7^ PFU, University of Iowa). Five months after inhalation, tumour formation was monitored bi-weekly by magnetic resonance imaging (MRI) (A 3.0 T Philips Achieva clinical MRI (Philips Best, the Netherlands) in combination with a dedicated mouse solenoid coil (Philips Hamburg, Germany), were used for imaging. T2-weighted MR images were acquired in the axial plane using turbo-spin echo (TSE) sequence [repetition time (TR) = 3819 ms, echo time (TE) = 60 ms, field of view (FOV) = 40 × 40 × 20 mm^3^, reconstructed voxel size = 0.13 × 0.13 × 1.0 mm^3^, number of average = 1) under isoflurane (2.5%) anaesthesia. MR images (DICOM files) were analysed blindly by determining and calculating region of interests (ROIs) using Horos software. Horos is a free and open source code software (FOSS) program that is distributed free of charge under the LGPL license at Horosproject.org and sponsored by Nimble Co LLC d/b/a Purview in Annapolis, MD, USA. Once tumours reached a minimum volume >1 mm^3^, mice were randomised into two groups and treated with either the vehicle HP-β-CD 10% (Hydroxypropyl Beta Cyclodextrin; #C0926-5G Sigma) or dinaciclib at 30 mg/kg (MedChemExpress, #HY-10492, Lot#120243) twice per week, every 2 weeks, until tumours reached a size of 800 mm^3^, at which point mice were sacrificed.

#### Syngeneic mouse model by subcutaneous injection of SCLC cells

424.3 cell line: 5 × 10^6^ cells in 100 µl PBS were injected subcutaneously into the flanks of 8-week-old female C57BL/6N mice (Charles River, Wilmington, MA, USA). Mice were randomly enroled either into vehicle or treatment groups once tumours reached a minimum size of 2 × 2 mm. Tumours were measured blindly using a calliper, and volume was calculated using the following formula: π/6×length×width^2^, where length is measured perpendicular from the width, and length>width. Treatment was carried out twice a week, every other week, until endpoint criteria were met. Treatment consisted of dinaciclib (Insight Biotechnology, #HY-10492, Lot#14761 and Lot#120243) at a dose of 20 mg/kg, in 10% HP-β-CD (Hydroxypropyl Beta Cyclodextrin; #C0926-5G Sigma).

1380 cell line: 3 × 10^6^ cells in 100 µl of Matrigel (Corning, Corning, NY, USA, cat# 354234) were injected subcutaneously into the flanks of 8-week-old female C57BL/6N mice obtained from the core facility of the CECAD research centre (Fig. 5A, B, Supplementary Fig. 5d), or 16-week old female C57BL/6 obtained from a specific-pathogen-free (SPF) colony of the Department of Experimental Pharmacology, National Institute of Oncology (Budapest, Hungary). Mice were randomly assigned to vehicle or treatment groups 10 days after the cell injection when the average tumour size reached 50–100 mm^3^. Treatment and tumour measurement was carried out as mentioned before for dinaciclib (MedChemExpress, #HY-10492, Lot#14761, and Lot#120243) at a dose of 30 mg/kg, in 10% HP-β-CD (Hydroxypropyl Beta Cyclodextrin; #C0926-5G Sigma). For VC-1, treatment consisted of three i.p. injections per week at a dose of 20 mg/kg. VC-1 was resuspended in miliQ water along with 10% HP-β-CD (Hydroxypropyl Beta Cyclodextrin; #CY2005.2 Cyclolab, Budapest, Hungary).

All animal experiments were conducted in compliance with international and institutional ethical guidelines on animal welfare and measures to minimise animal suffering.

### VC-1 synthesis

2-methoxybenzoyl chloride (1) was reacted with potassium isothiocyanate and urea to form N-[(carbamoylamino)carbonothioyl]-2-methoxybenzamide (3). 3 was cyclisized by 40% aq. NaOH at room temperature in aqueous media to give 6-(2-methoxyphenyl)-4-thioxo-3,4-dihydro-1,3,5-triazin-2(1H)-one (4) then –SH was alkylated by methyl-iodide to form 4-(2-methoxyphenyl)-6-(methylthio)-1,3,5-triazin-2-ol (5). Compound 5 was chlorinated with thionyl-chloride forming 2-chloro-4-(2-methoxyphenyl)-6-(methylthio)-1,3,5-triazine (6) then 6 was coupled with 3-(1H-benzimidazol-1-ylmethyl)aniline in tBuOH in a presence of hydrogen-chloride to give N-[3-(1H-benzimidazol-1-ylmethyl)phenyl]-4-(2-methoxyphenyl)-6-(methylthio)-1,3,5-triazin-2-amine (7). In the final synthetic step, S-Me was cleaved by Raney-Nickel under hydrogen atmosphere to form VC-1. The crude product was purified by column chromatography. (Kieselgel, chloroform/methanol 20:1, as solvent). Synthesis illustrated in Supplementary Fig. 4d.

Molecular Weight: 444.46.

### VC-1 affinity determination

CDK9/CycT1 kinase assays were performed in low protein binding 384-well plates (Corning 3676). Test compounds were diluted in 100% DMSO to 5 mM stock concentration, and then further dilutions were made in 100% DMSO to desirable concentrations. Each reaction consisted of 5 nM enzyme: CDK9/CyclinT1 (Proqinase, Freiburg, Germany, #0371-0345-1), 400 nM TAMRA-Rbtide (synthetic 15-mer peptide derived from human retinoblastoma tumour suppressor protein labelled with TAMRA dye, Genecust Europe, Boynes, France), 12 μM ATP (=Km_app_, Sigma-Aldrich) and kinase buffer: 20 mM MOPS pH 7 (Sigma-Aldrich), 1 mM DTT (Sigma-Aldrich), 10 mM MgCl_2_ (Sigma-Aldrich), 0.01% Tween 20 (Sigma-Aldrich). For each reaction, 4 or 6 μL containing TAMRA-Rb peptide, ATP, and kinase buffer were combined with 0.028 μL of VC-1 in 100% DMSO. The kinase reaction was started by adding 2 μL of the diluted enzyme. The reaction was allowed to run for 1 h at room temperature. The reaction was stopped by adding 10 μL of IMAP beads (1:400 beads in progressive (100% buffer A) 1× buffer). After an additional 1 h, fluorescent polarisation (Ex: 530-5 nm, Em: 590-20 nm) was measured using a Tecan Infinite M1000Pro reader. Measurements were used to determine the % of inhibition. IC_50_ curve was fitted with XLfit 5.1.0.0 (IDBS, Woking, UK) curve fitting software.

### Residual kinase activity assay

VC-1 compound was profiled at ProQinase GmbH using a proprietary selectivity assay. The kinase inhibition profile of VC-1 was determined by measuring residual activity values at a single concentration in duplicate in 16 protein kinase assays. Results were provided by the company and reported in the paper (Fig. 4C).

### Statistical analyses

Statistical analyses were carried out using GraphPad Prism 8 v8.0.2. (GraphPad Software Inc., San Diego, CA, USA) IC_50_s were calculated by non-linear regression comparing normalised response (null hypothesis) vs. normalised response – variable slope and choosing the better model in each case. Log-Rank (Mantel–Cox) analysis was used to compare survival.

### Supplementary information

Supplementary Figures

Western Blot full films

## Data Availability

Data sharing not applicable to this article as no datasets were generated or analysed during the current study.
