# Supplementary material for: CDK9 inhibition as an effective therapy for small cell lung cancer
Source: Cell Death Dis. 2024 May 20;15(5):345. doi: 10.1038/s41419-024-06724-4 (PMC11106072; doi:10.1038/s41419-024-06724-4)
Supplement: Supplementary file 1 — Supplementary Figures [file 41419_2024_6724_MOESM1_ESM.pdf]

Supplementary Figure 1

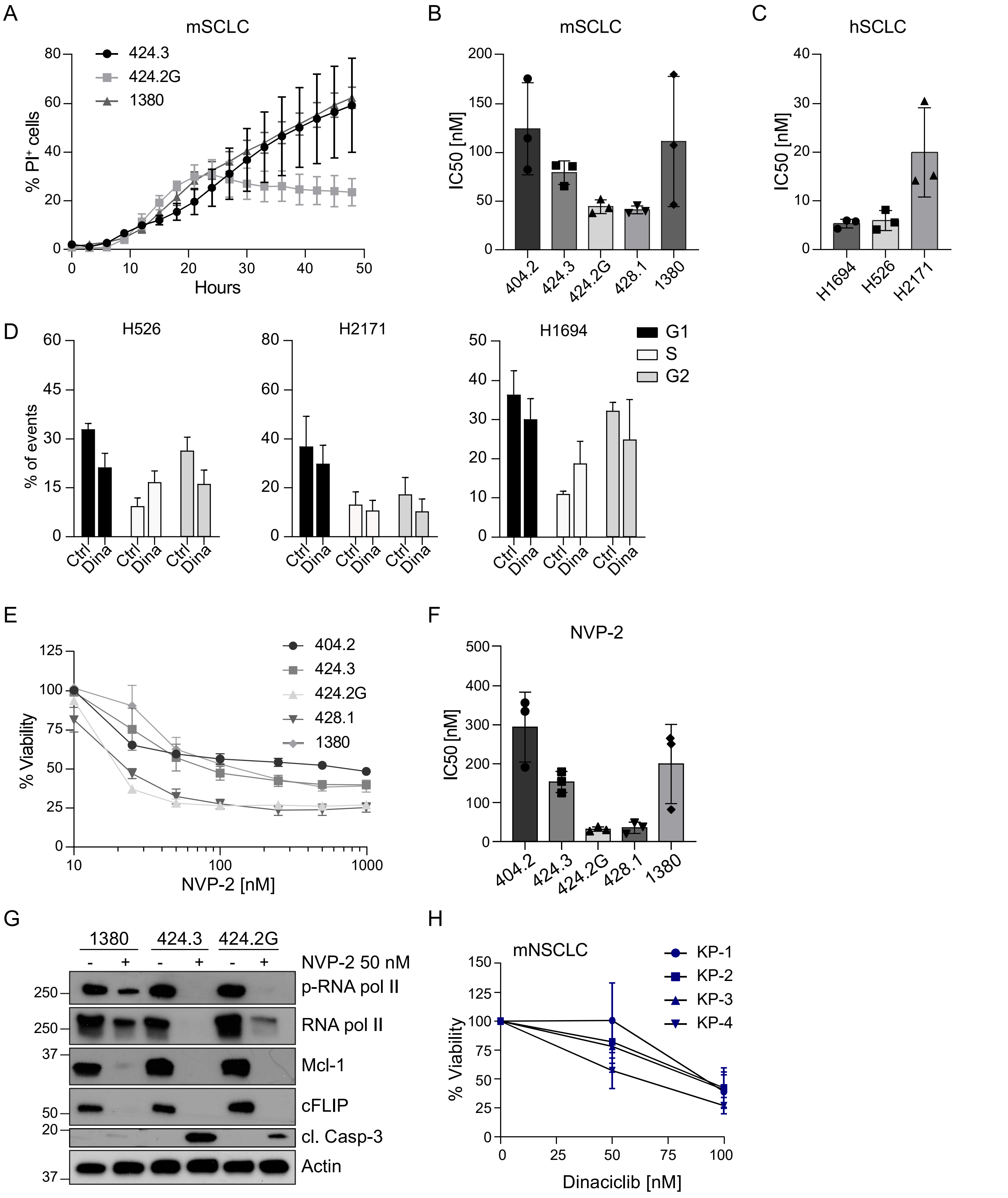

Supplementary Fig. 1: Mouse and human SCLC and NSCLC cells show different sensitivities to dinaciclib and NVP-2

A) Percentage of PI-positive cells after treatment with 50 nM dinaciclib as measured by Incucyte with Cell-by-cell analysis. Mean + SD, n=3. B) IC_{50} (nM) of mouse SCLC. Mean + SD, n=3. C) and human SCLC cell lines. Mean + SD, n=3. D) Cell cycle distribution was assessed by permeabilising the cells and staining with PI after 24 hours of treatment with 50 nM of dinaciclib. Mean +SD, n=3. Two-way ANOVA. Dunnet's multiple comparison test *p-adj < 0.05. E) Viability, as measured by CTG, expressed as percentage of the untreated control (100%) after a 30-hour treatment with different concentrations of NVP-2 (10, 25, 50, 100, 250, 500 and 1 000 nM). Mean + SD, n=3. F) IC_{50} of NVP-2 for each cell line. Mean + SD, n=3. G) Cells were lysed with RIPA buffer after 30 hours of treatment with NVP-2 (50 nM) or vehicle. Representative blots of 3 independent experiments. p- = phospho; cl. = cleaved. H) Viability of mouse NSCLC cell lines after a 30-hour treatment at 50 nM and 100 nM of dinaciclib. Mean + SD, n=3.

Supplementary Figure 2

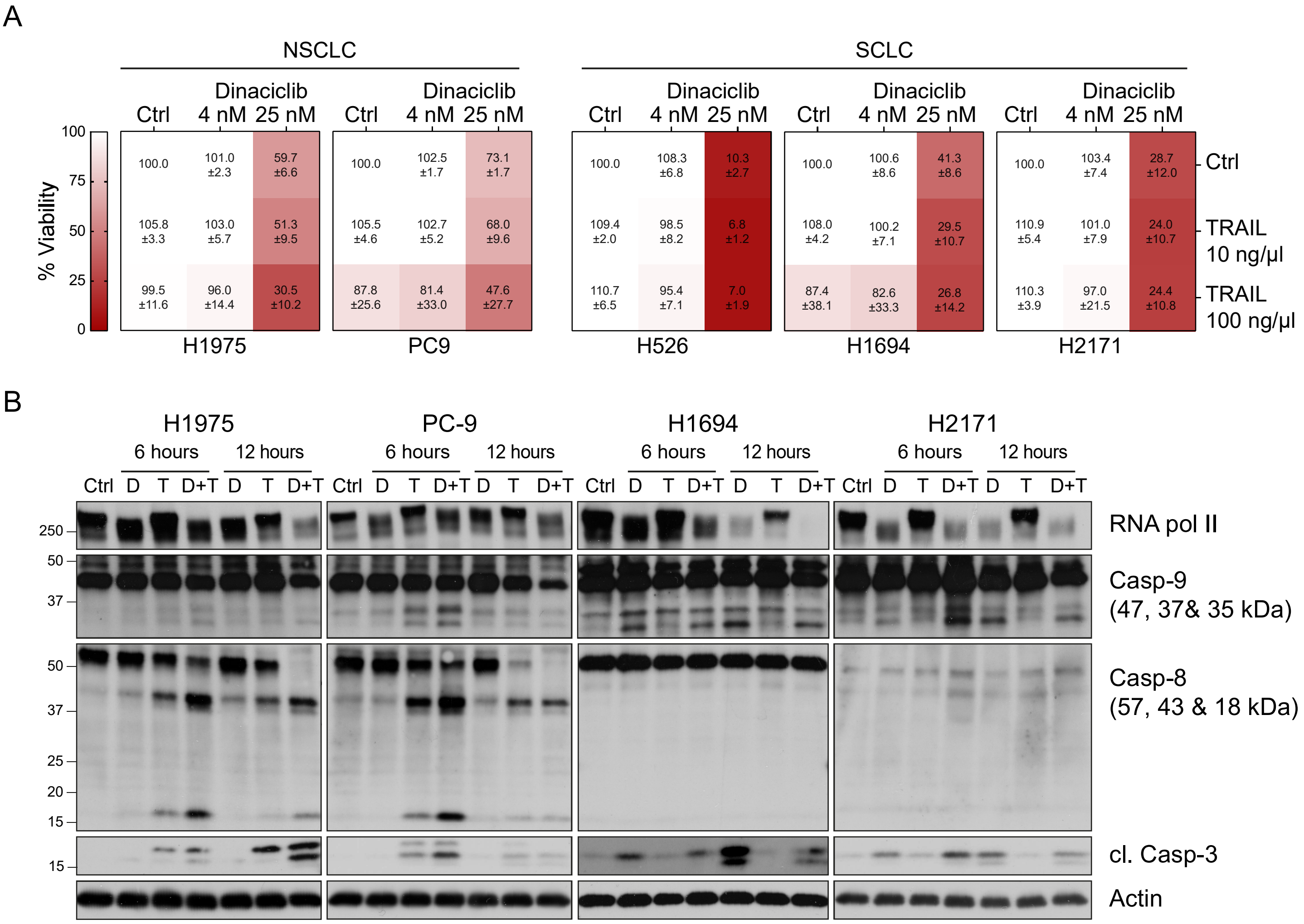

Supplementary Fig. 2: CDK9 inhibition shows no synergy with TRAIL in SCLC

A) Viability, as measured by CTG, expressed as a percentage of the untreated control (100%) after a 30-hour treatment with dinaciclib (4 nM, 20 nM) and TRAIL (10 ng/µl, 100 ng/µl). Mean + SD of at least 3 independent experiments. B) Cells were lysed with RIPA buffer after 6 and 12 hours of treatment with 50 nM dinaciclib and/or 100 ng/µl of TRAIL. Representative blots of 3 independent experiments.

Supplementary Figure 3

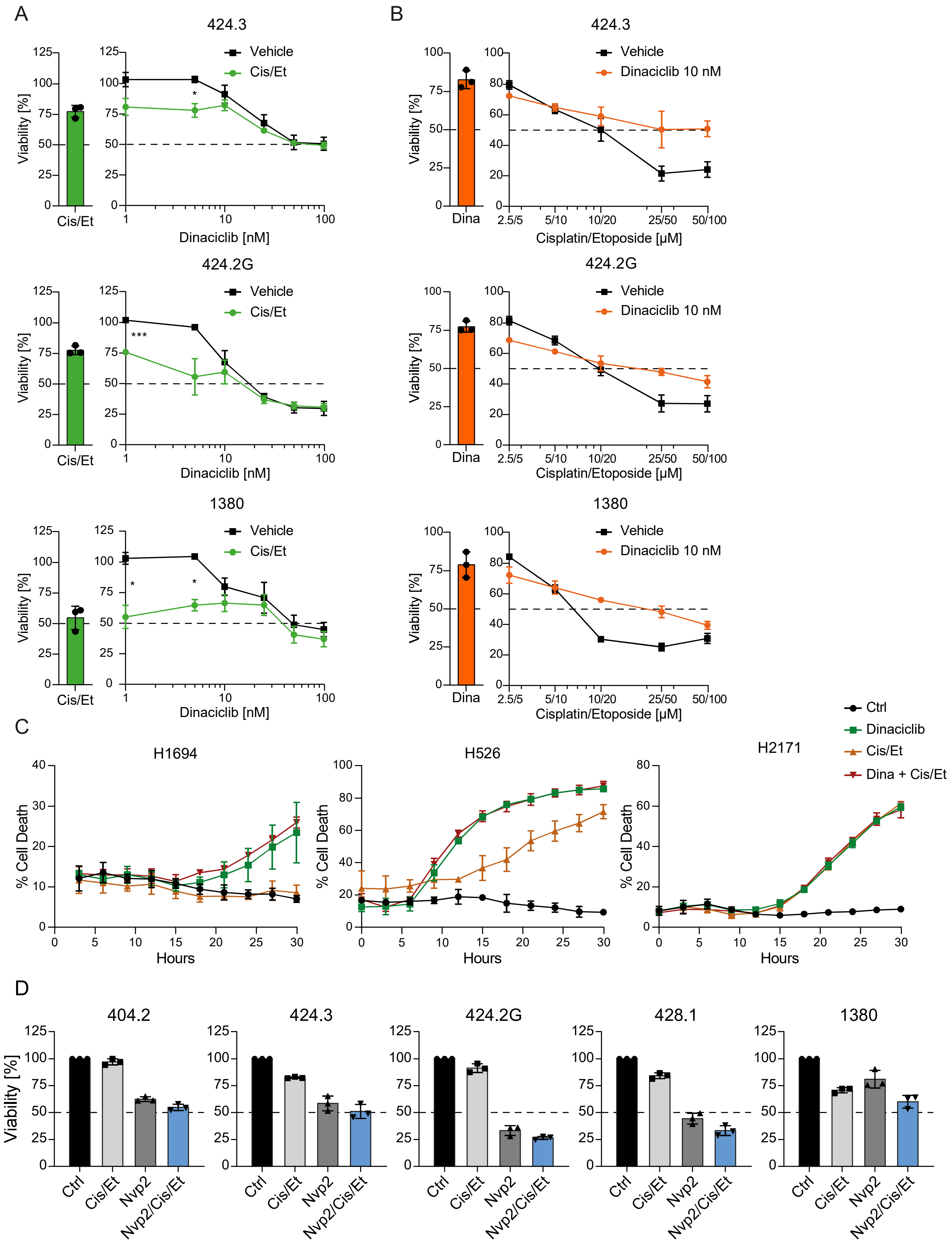

Supplementary Fig. 3: CDK9 inhibition shows no synergy with standard chemotherapy in SCLC

A) Mouse SCLC cells were treated with increasing doses of dinaciclib (1, 5, 10, 25, 50 and 100 nM) for 30 hours in the presence or absence of a combination of cisplatin (5 μM) and etoposide (10 μM). Mean + SD, n=3. B) Mouse SCLC cells were treated with increasing doses of cisplatin and etoposide for 30 hours in the presence or absence of 10 nM dinaciclib. Mean + SD, n=3. C) Percentage of PI-positive cells as measured by Incucyte after treatment with 25 nM dinaciclib and/or cisplatin & etoposide. H526 & H1694: Cis 1.25 μM, Et 3.125 μM. H2171: Cis 40 μM, Et 100 μM. Mean + SD. Representative graphs of 3 independent experiments. D) Mouse SCLC cells were treated with 25 nM NVP-2, 5 μM cisplatin and/or 10 μM etoposide for 30 hours. Viability was measured by CTG and expressed as a percentage of the viability of control. Mean + SD, n=3..

Supplementary Figure 4

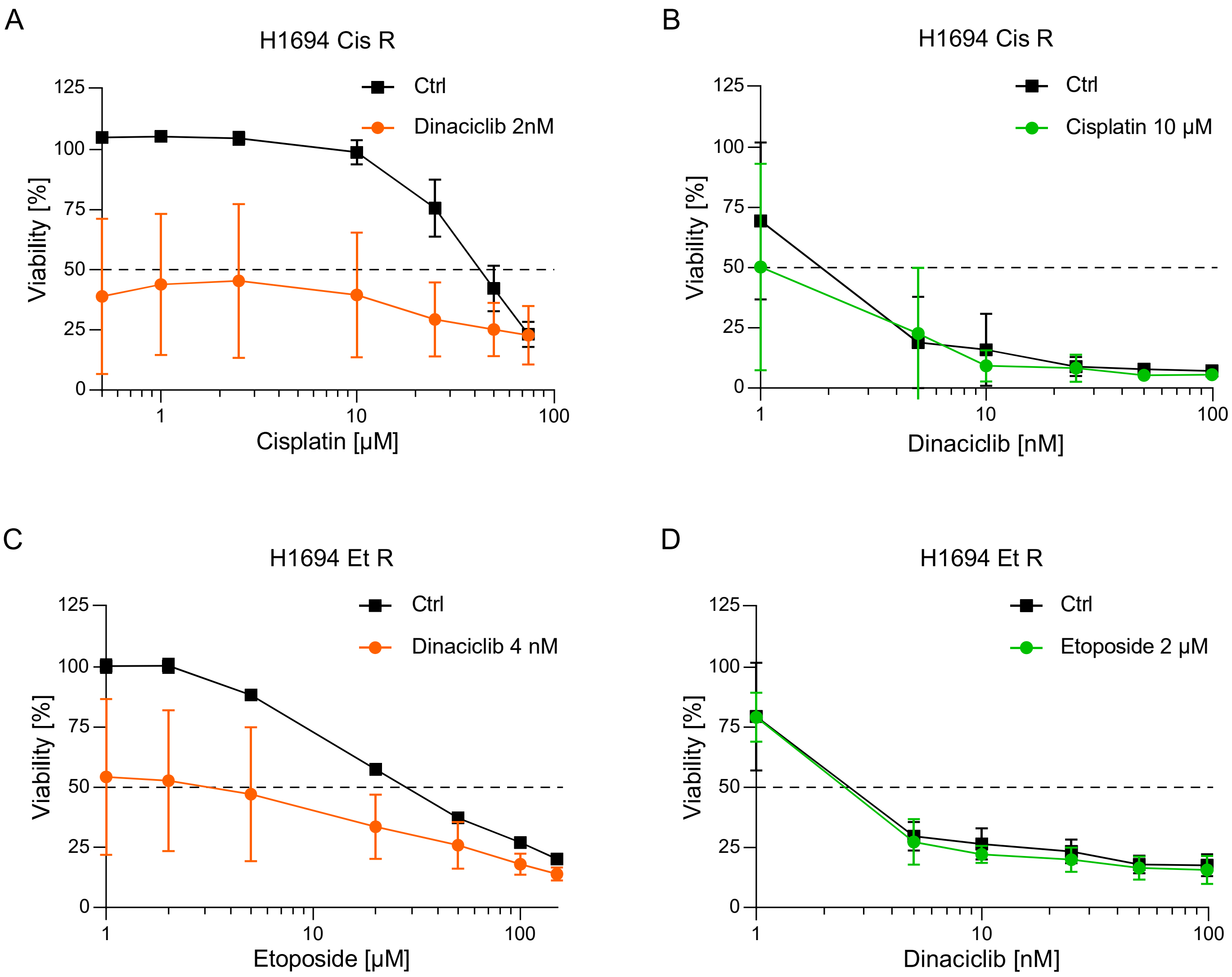

Supplementary Fig. 4: Dinaciclib treatment efficiently kills SCLC cells with acquired resistance to chemotherapy

A) and B) SCLC cells with acquired resistance to cisplatin (Cis R) were treated with increasing doses of cisplatin (0.5, 1, 2.5,10, 25, 50 and 75 µM) for 30 hours with or without dinaciclib 2nM (A), or increasing doses of dinaciclib (1, 5, 10, 25, 50, and 100 nM) with or without cisplatin 10 µM (B). C) and D) SCLC cells with acquired resistance to etoposide (Et R) were treated with increasing doses of etoposide (1, 2, 5, 20, 50 and 100 µM) for 30 hours with or without dinaciclib 4nM (C), or increasing doses of dinaciclib (1, 5, 10, 25, 50, and 100 nM) with or without etoposide 2 µM (D). Viability was measured by CTG and expressed as a percentage of the viability of control. Mean + SD, n=3.

Supplementary Figure 5

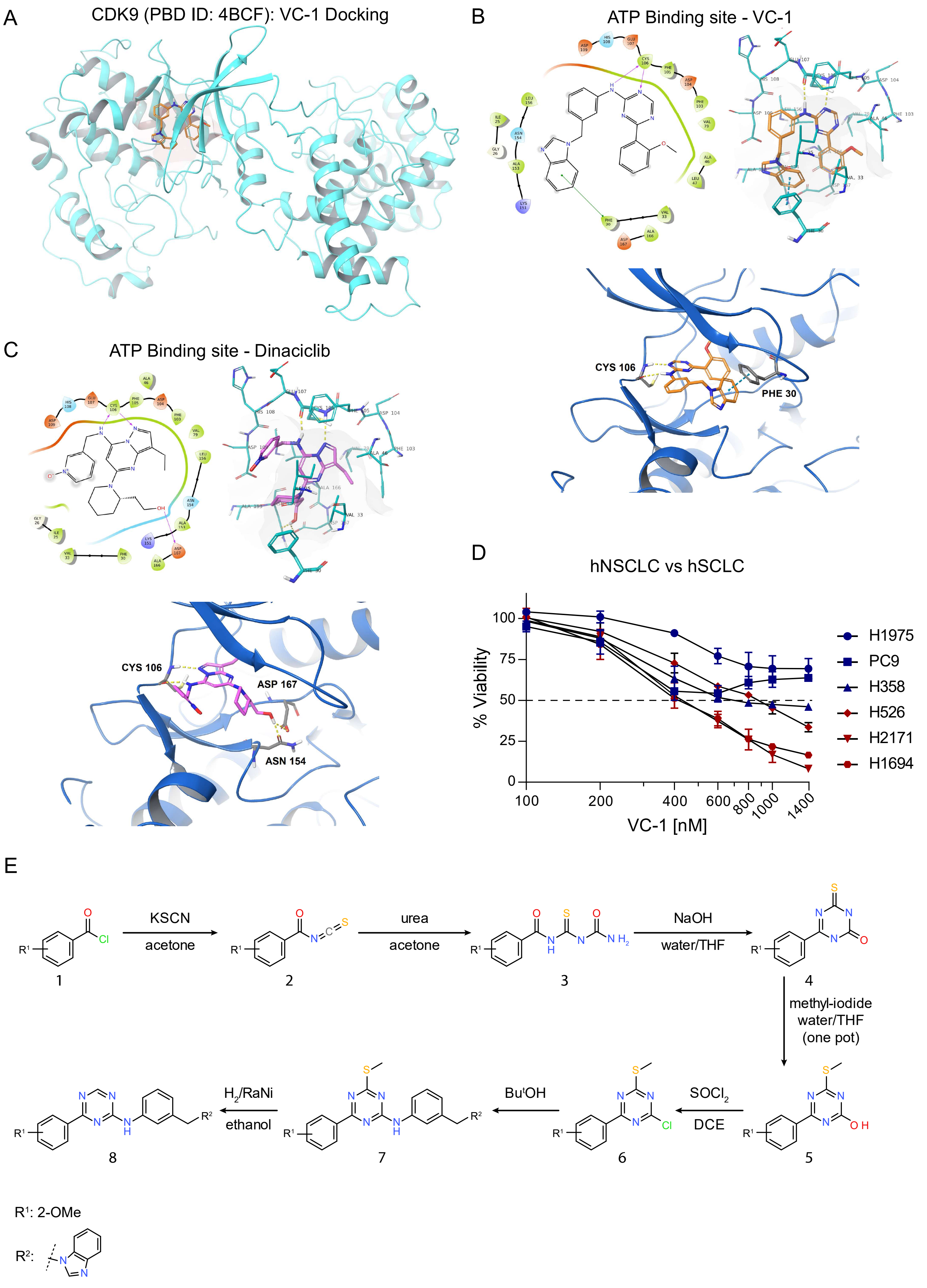

Supplementary Fig. 5: A new class of CDK9 inhibitor

A) Crystal structure of CDK9 (PBD ID: 4BCF) modelling VC-1 in its ATP binding site. Illustrations and model of interactions of VC-1 (B) and Dinaciclib (C) within the ATP binding site of CDK9. E) Step-by-step synthesis of VC-1, as stated in materials and methods. D) Viability was measured by CTG and expressed as a percentage of the viability of Control after a 30-hour treatment with different concentrations of VC-1 (100, 200, 400, 600, 800, 1 000 and 1 400 nM) Mean + SD, n=3.

Supplementary Figure 6

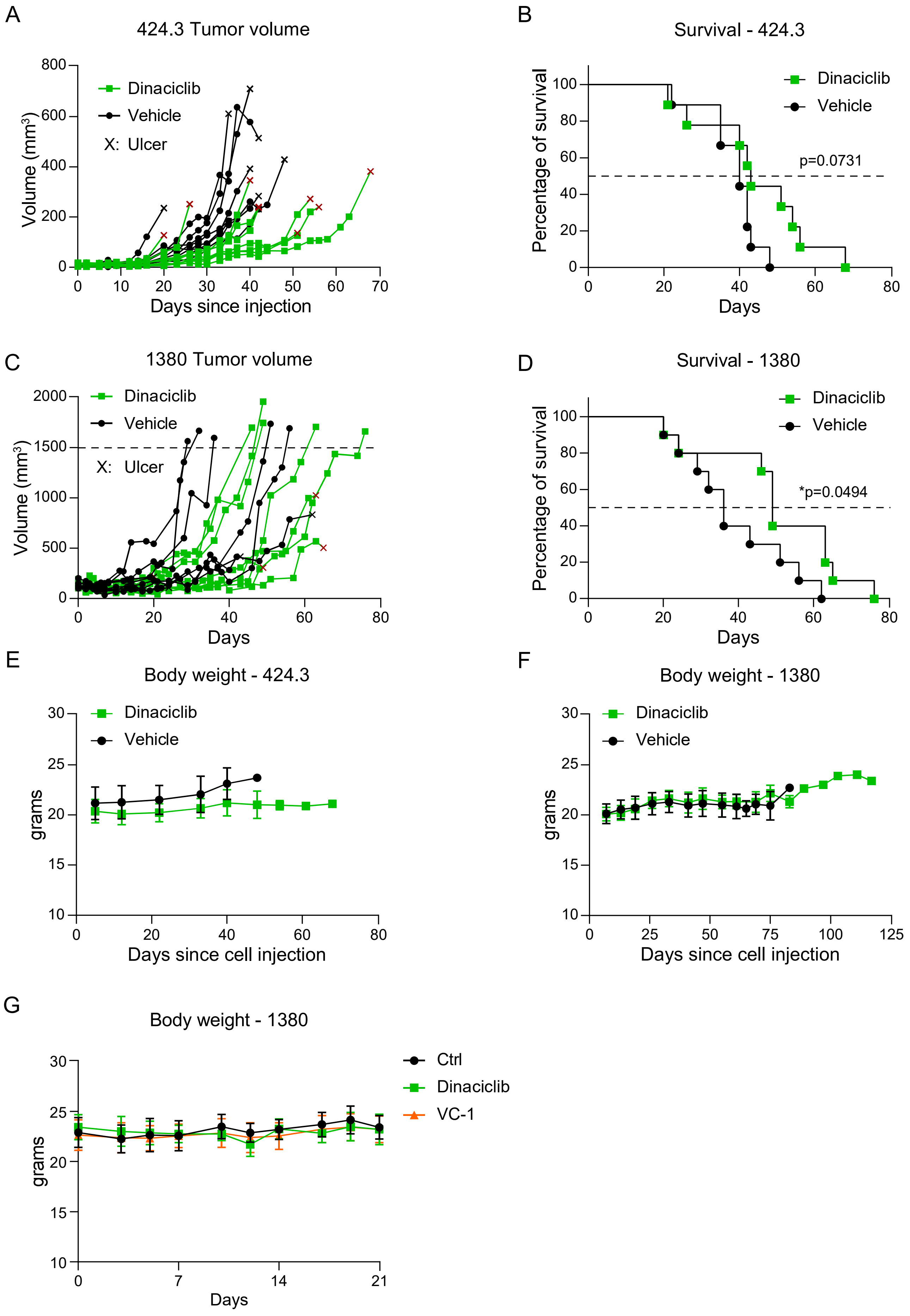

Supplementary Fig. 6: Dinaciclib reduces tumour growth and improves survival

A) 424.3 mouse cells were injected subcutaneously on the flank of C57BL/6 mice. Treatment began 16 days after injection with either dinaciclib (20mg/kg) or vehicle (10% Hydroxypropyl Beta Cyclodextrin) twice per week, followed by a week of drug holiday. Tumours were measured three times a week. N=9 per group. B) Survival curve of mice from supplementary Fig 6a. Log-rank (Mantel-Cox) test. C) 1380 mouse cells were injected subcutaneously on the flank of C57BL/6 mice. Treatment began upon tumour establishment with either dinaciclib (30mg/kg) or vehicle. Tumours were measured two times a week. N=10 per group. D) Survival curve of mice from supplementary Fig 6c. Log-rank (Mantel-Cox) test. *p = 0.0494. E) Weight of mice from supplementary Fig. 6a,b. Mean + SD. E) Weight of mice from supplementary Fig. 6c, d since injection with 1380 cells. Mean + SD. F) Body weight of mice treated as described in Fig. 5f, g from the first day of treatment. Mean + SD.
