# Supplementary material for: CDK9 inhibition as an effective therapy for small cell lung cancer
Source: Cell Death Dis. 2024 May 20;15(5):345. doi: 10.1038/s41419-024-06724-4 (PMC11106072; doi:10.1038/s41419-024-06724-4)

Figure 1D

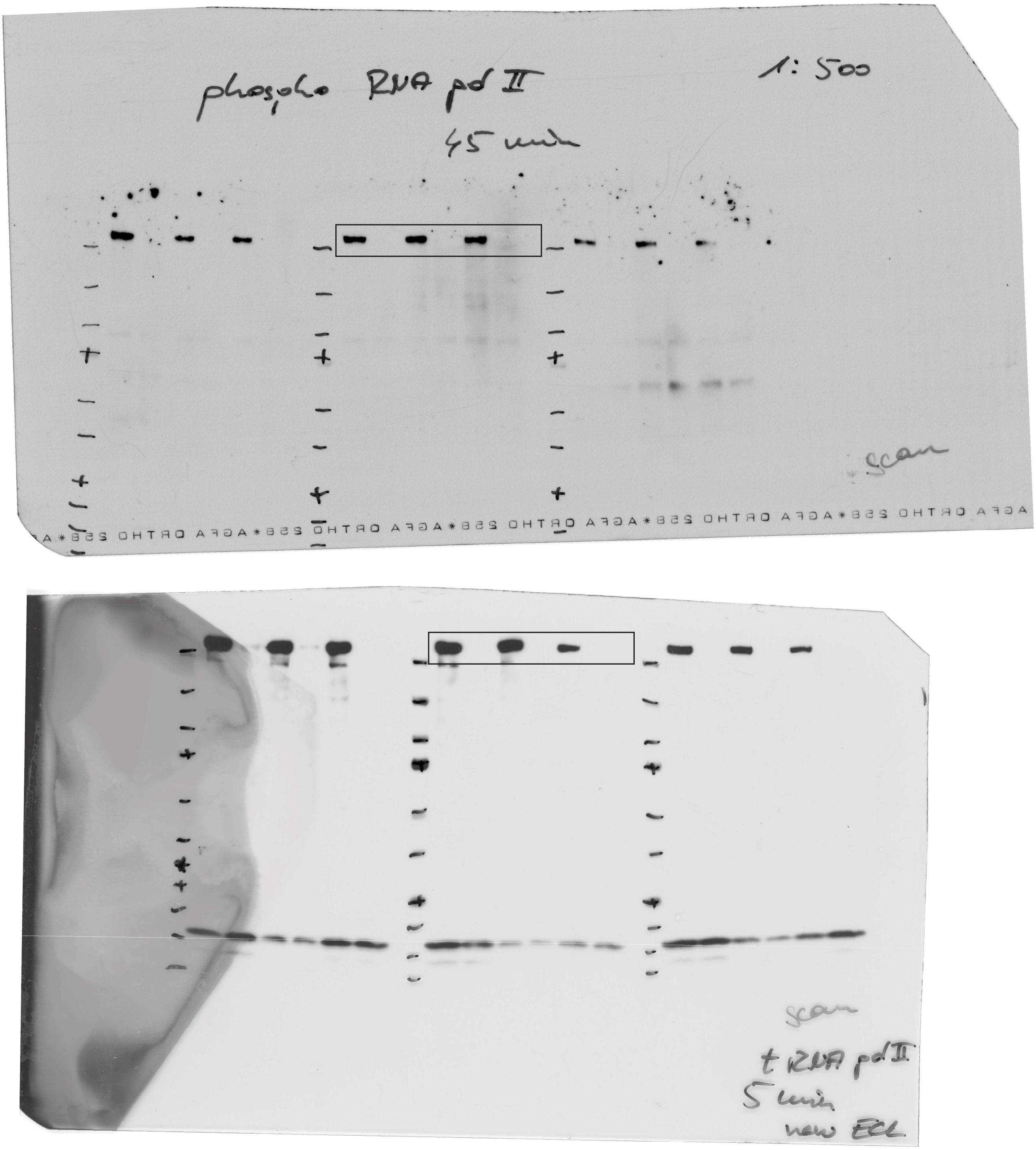
phospho RNA pol II
1:500
45 min
scan
scan
t RNA pol II
5 min
new ECL

Figure 1D

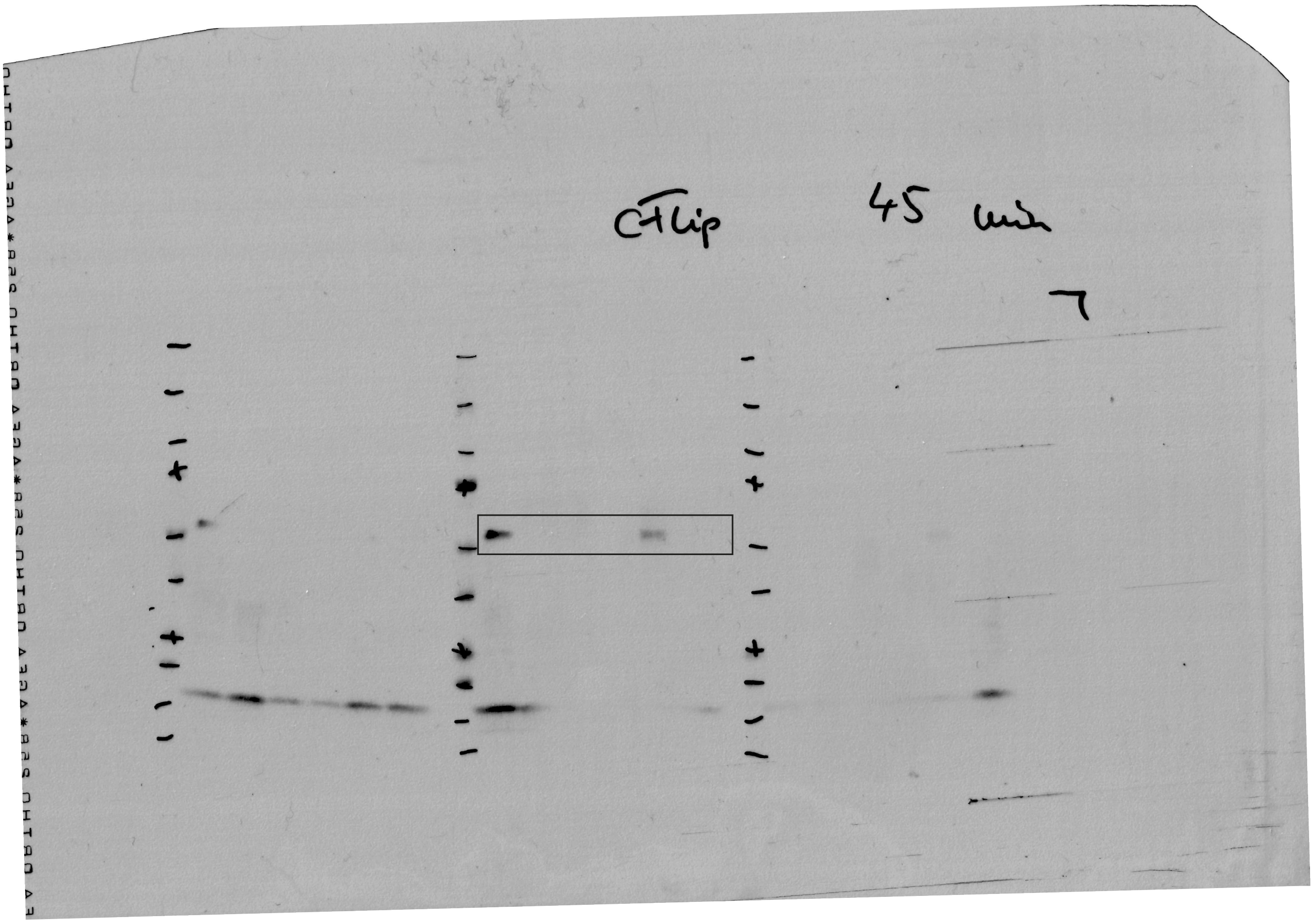

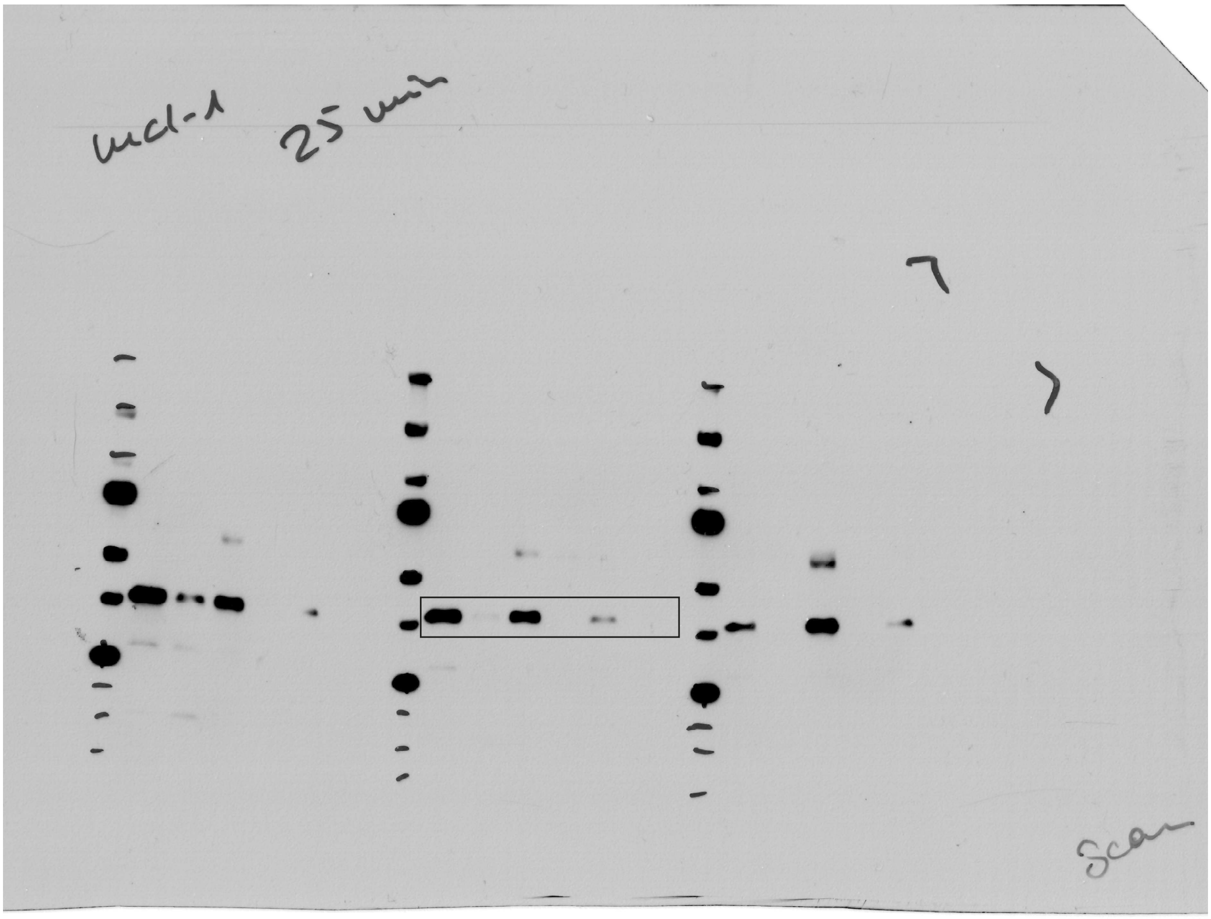

Figure 1D

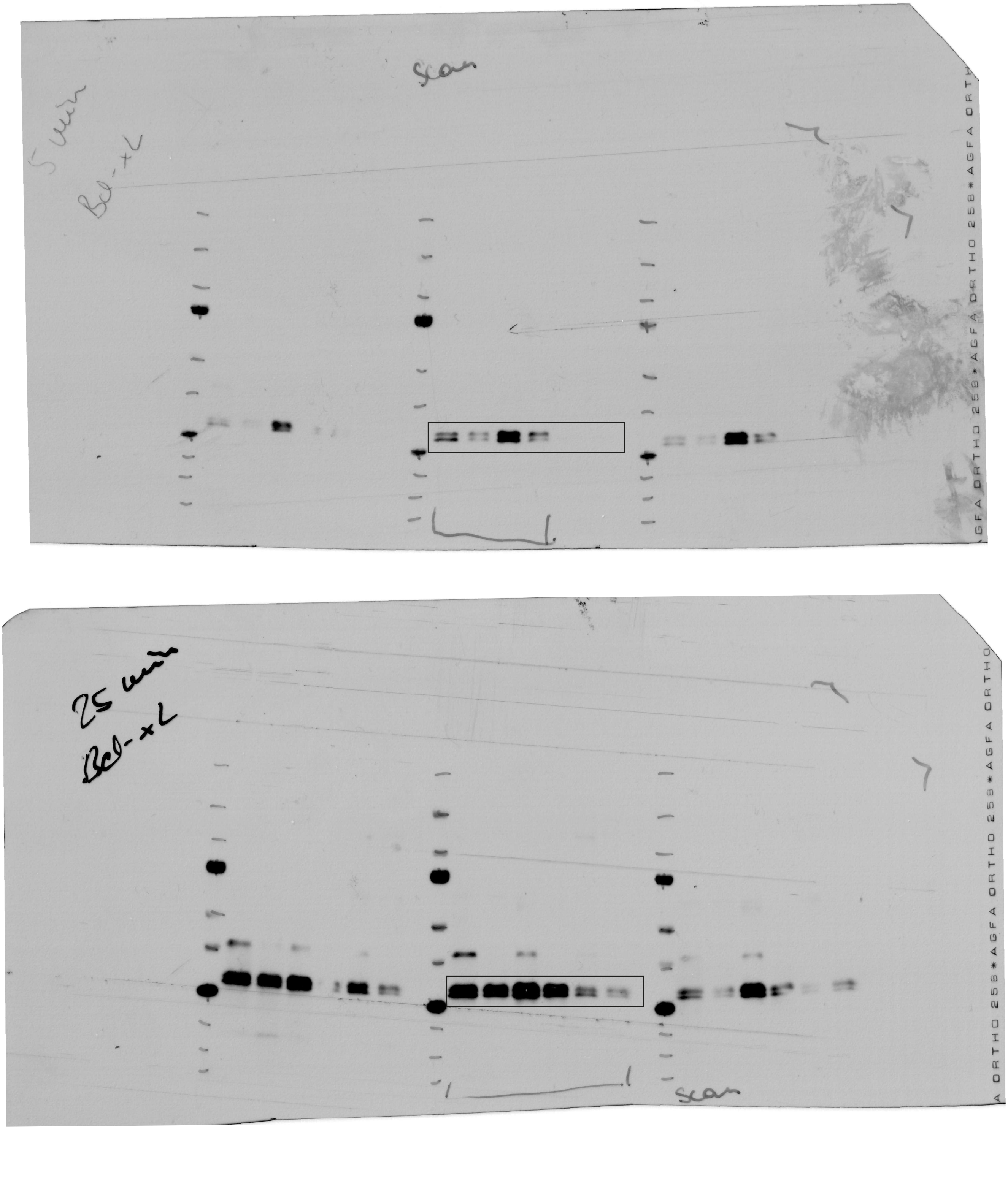

Figure 1D

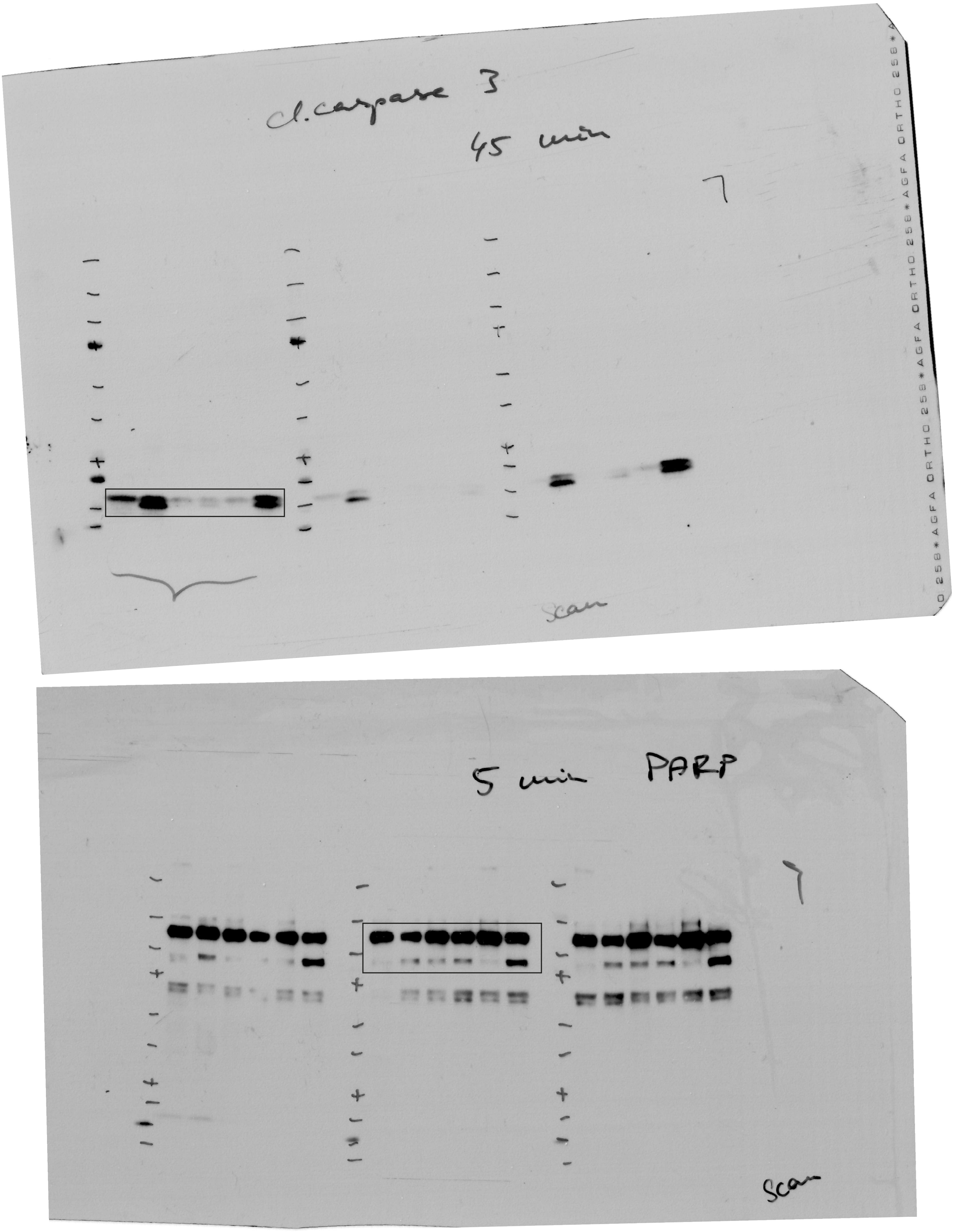

Figure 1D

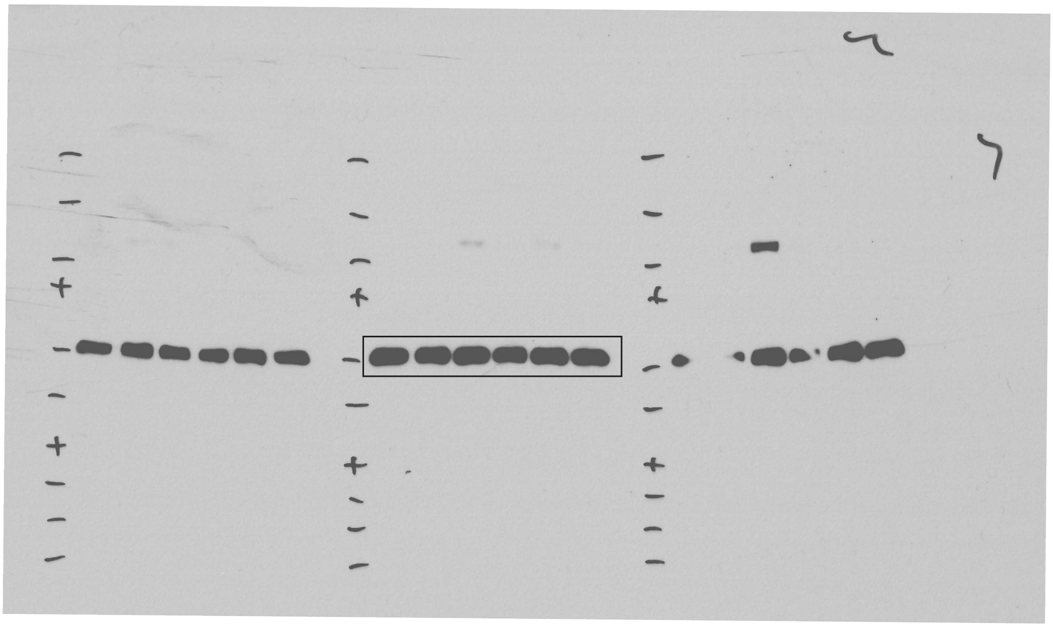

Figure 1E

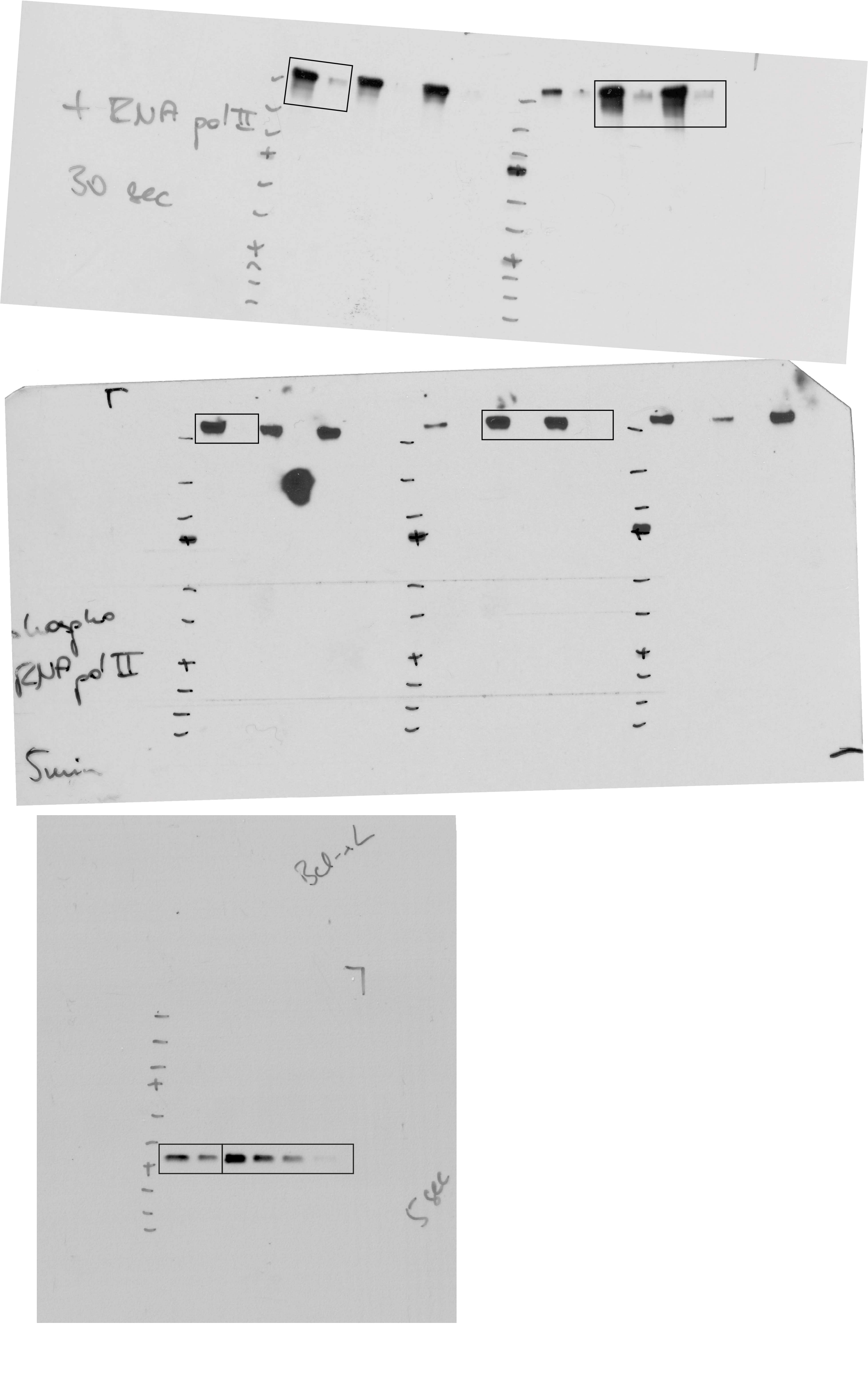

Figure 1E

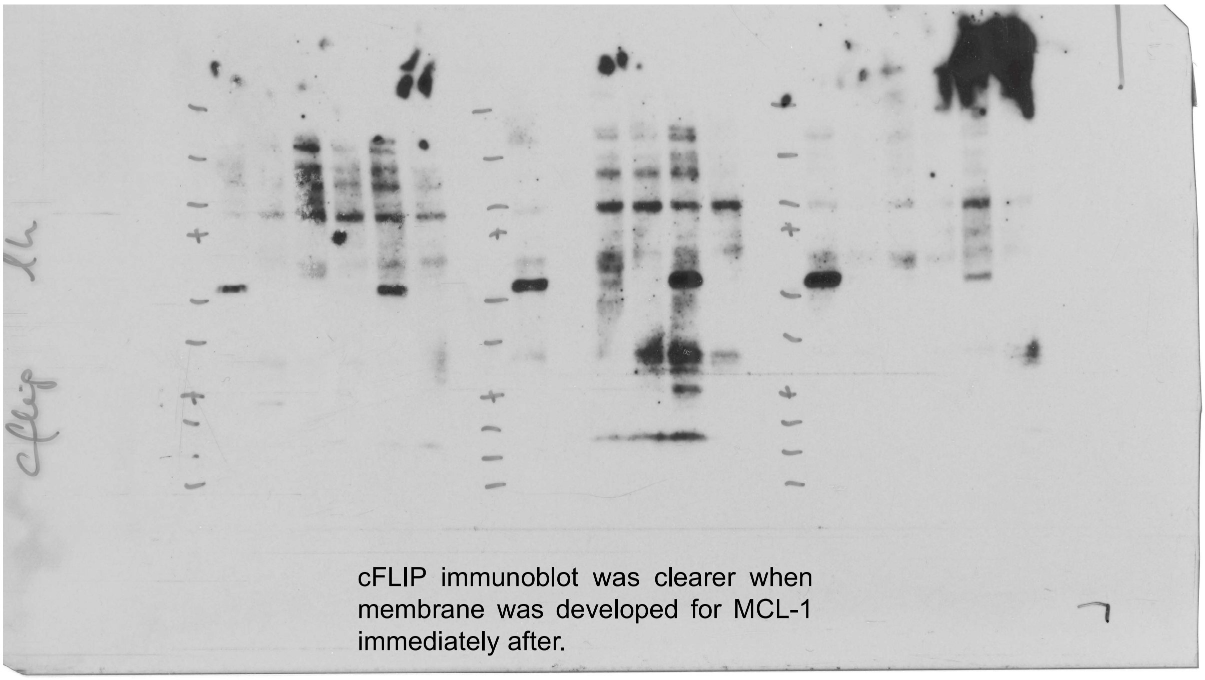

cFLIP immunoblot was clearer when membrane was developed for MCL-1 immediately after.

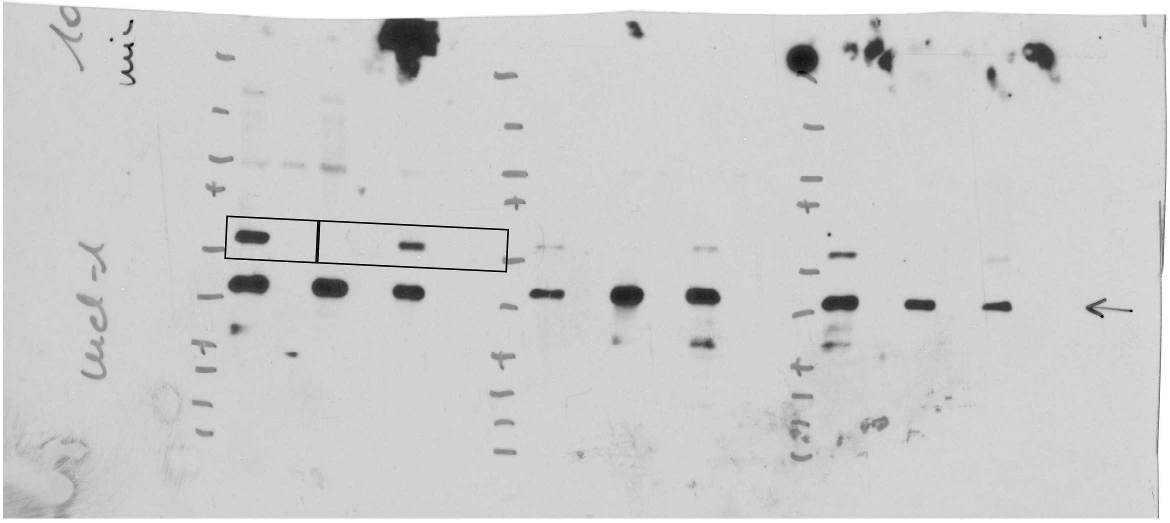

Figure 1E

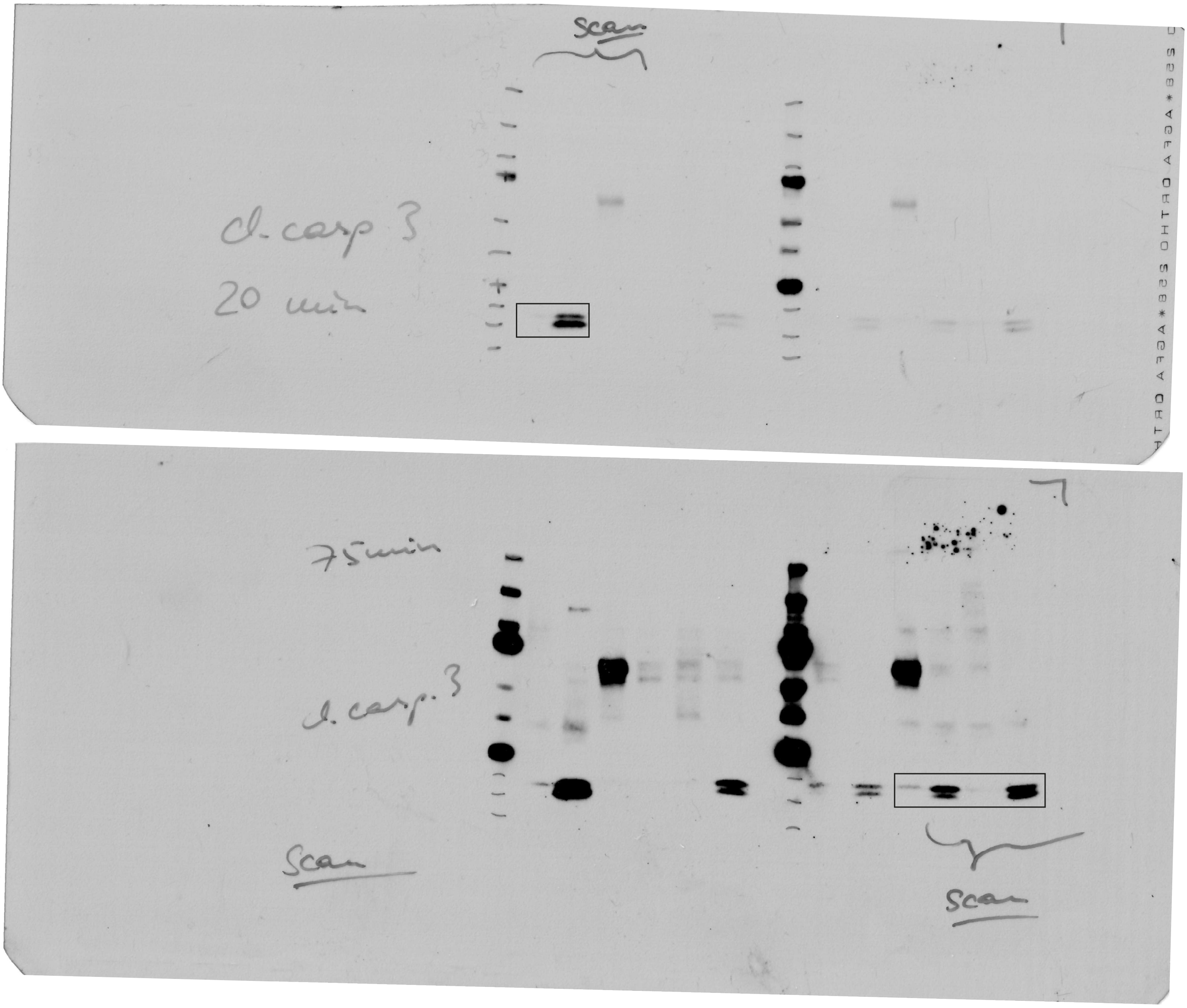

Figure 1E

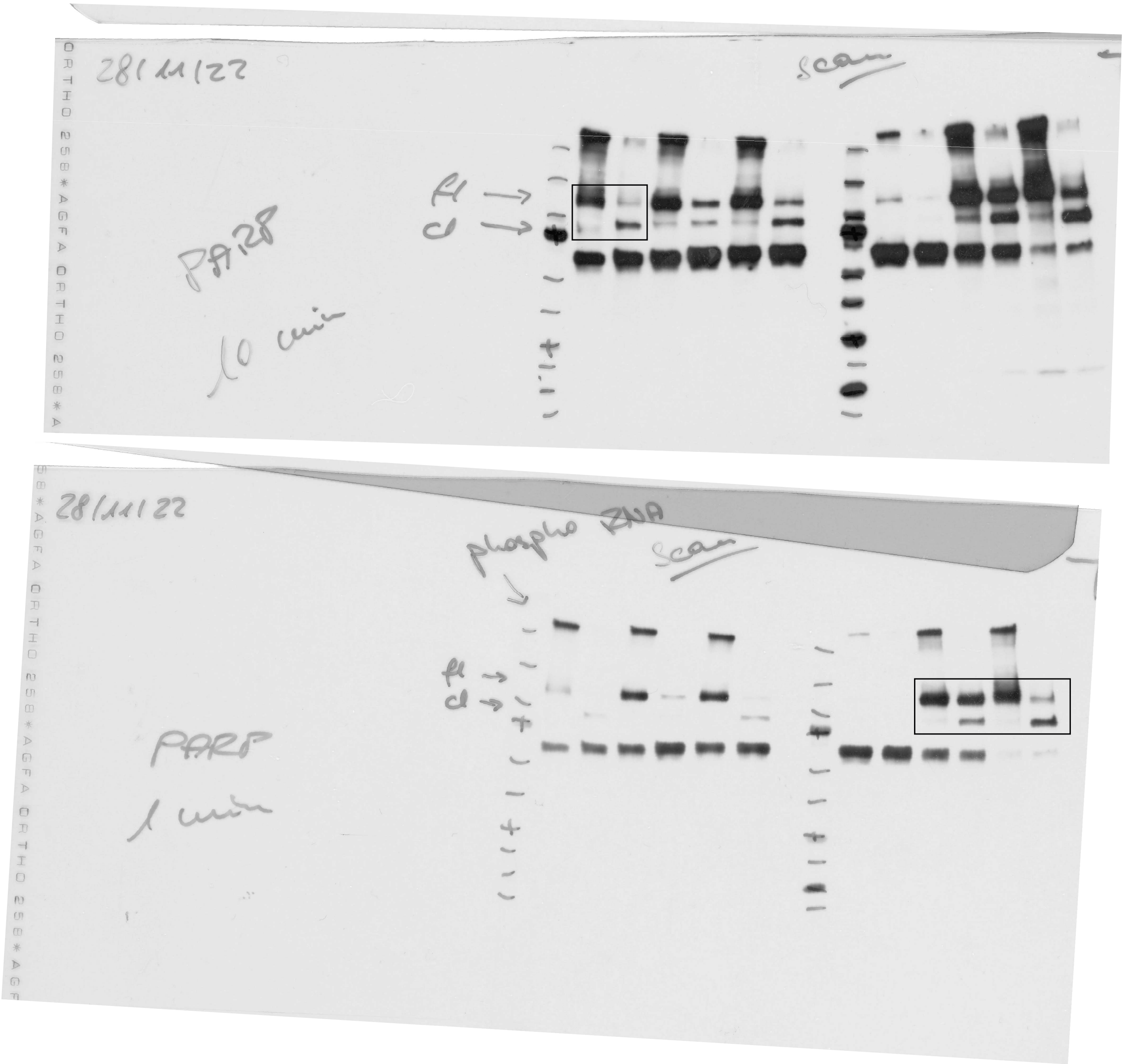
28/11/22
scan
PARP
10 min
fl
cl
28/11/22
phospho RNA
scan
fl
cl
PARP
1 min

Figure 1E

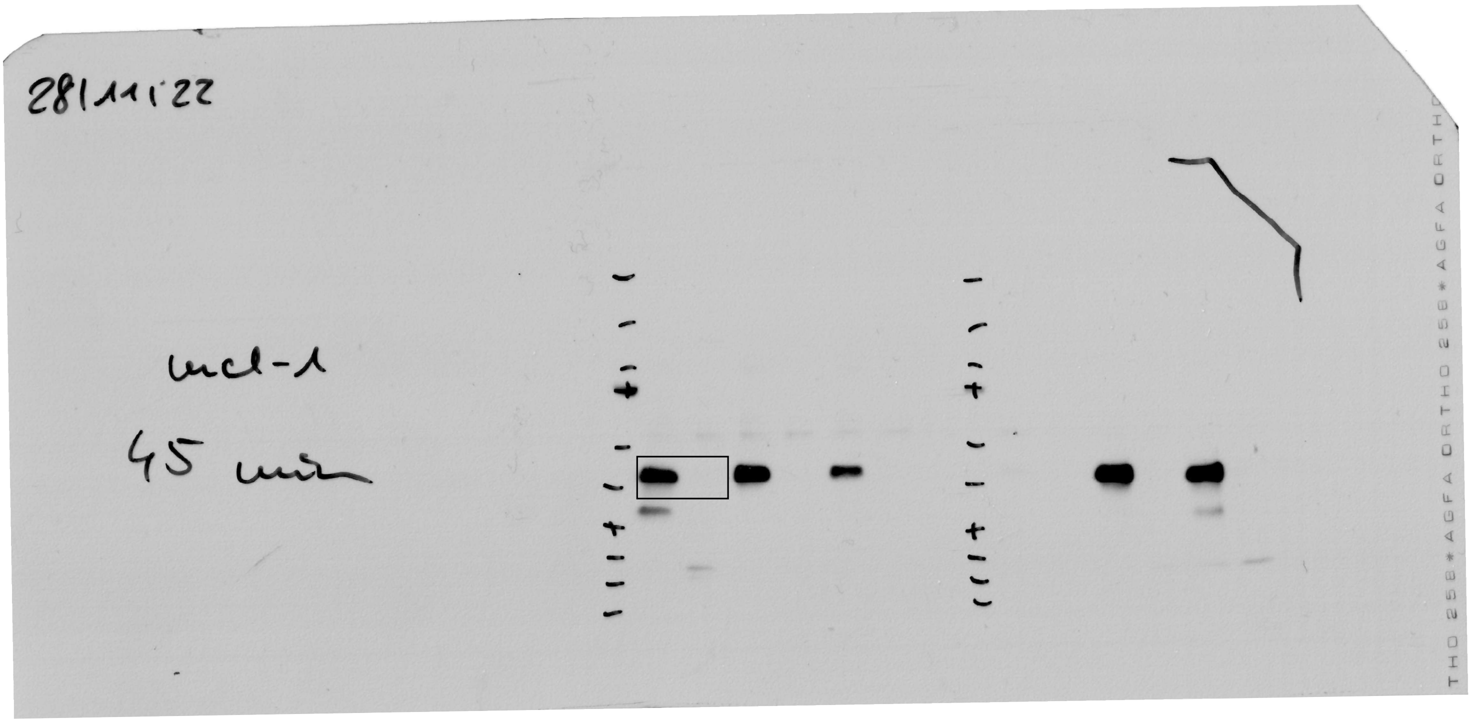

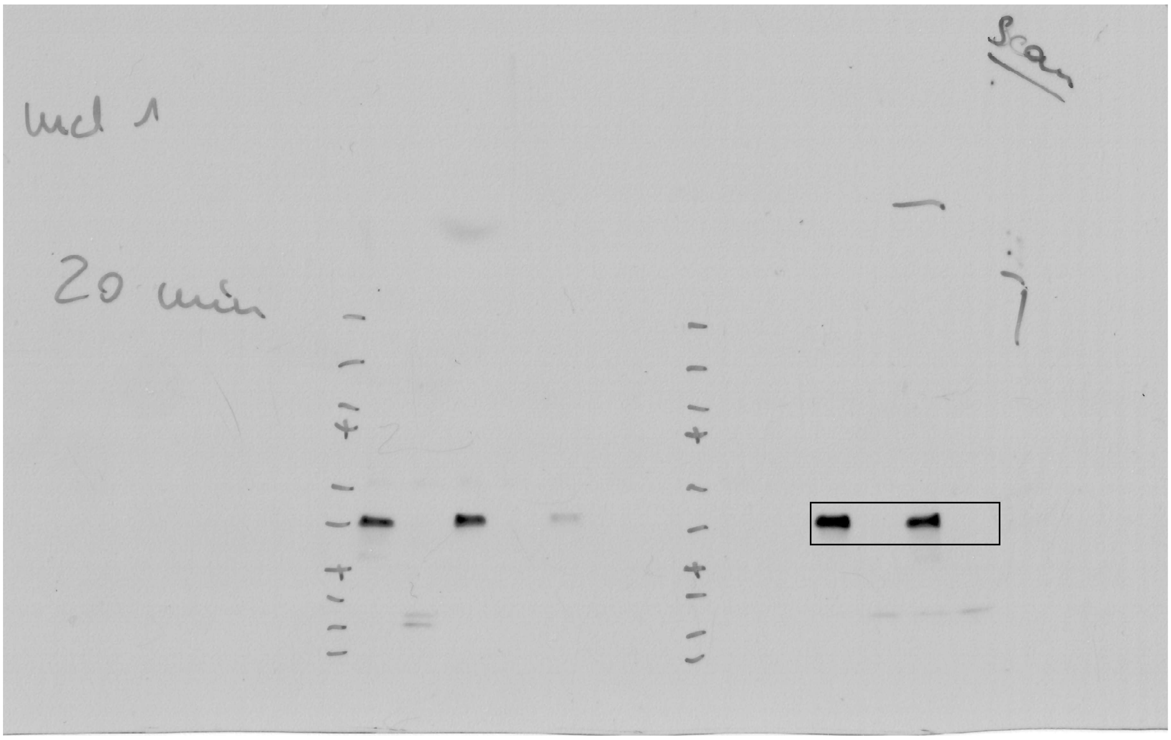

Figure 1E

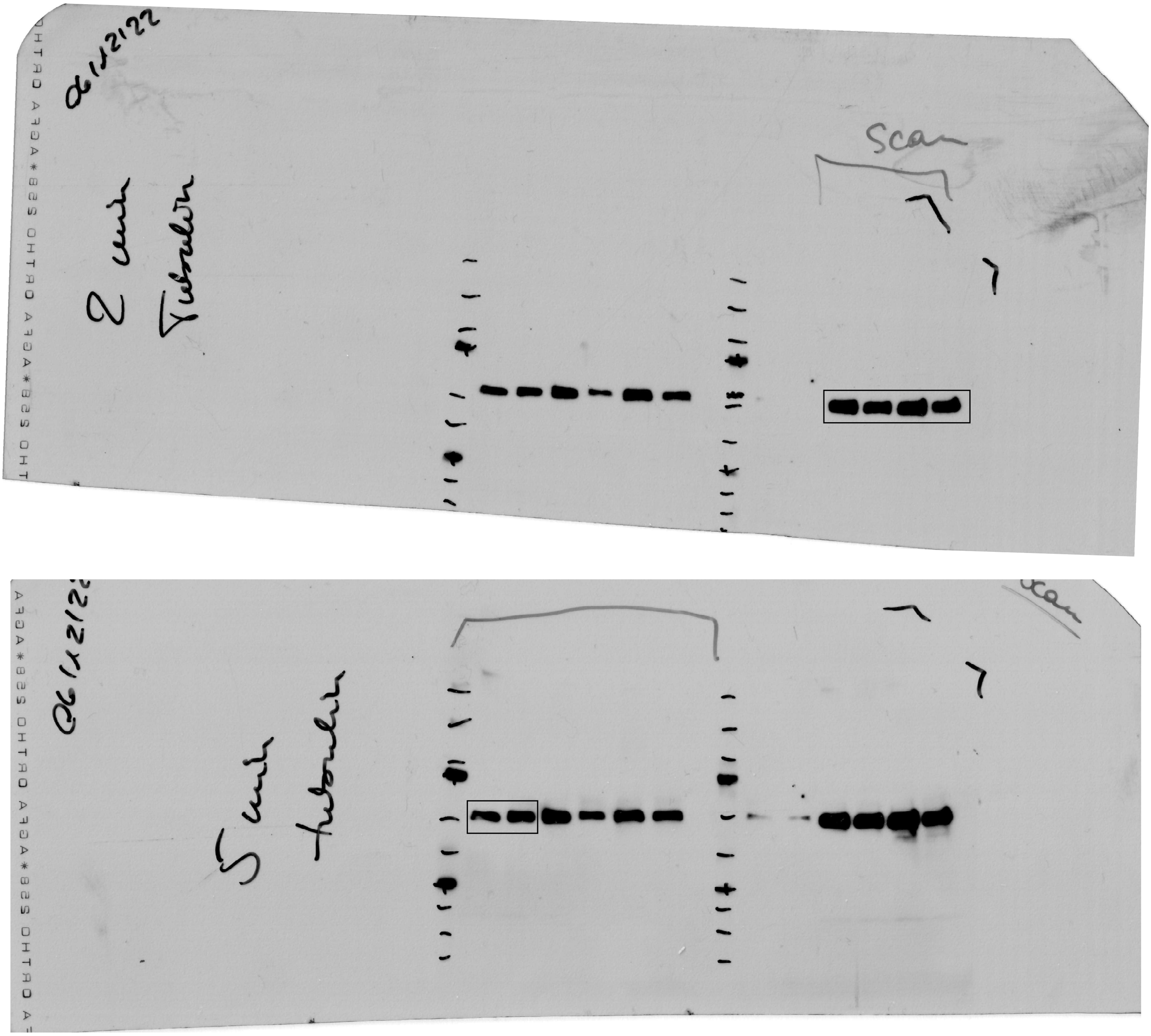

Figure 4G

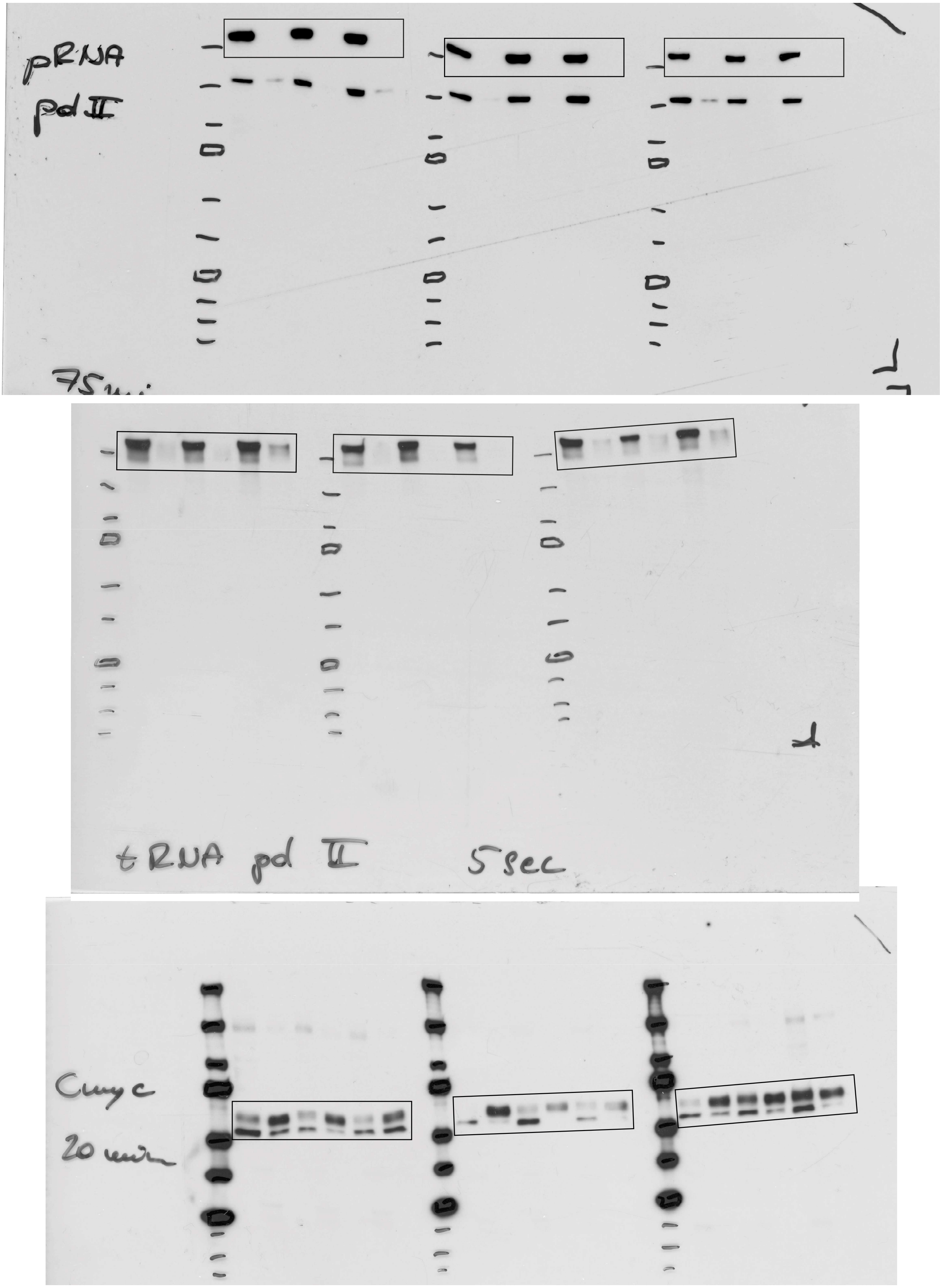

Figure 4G

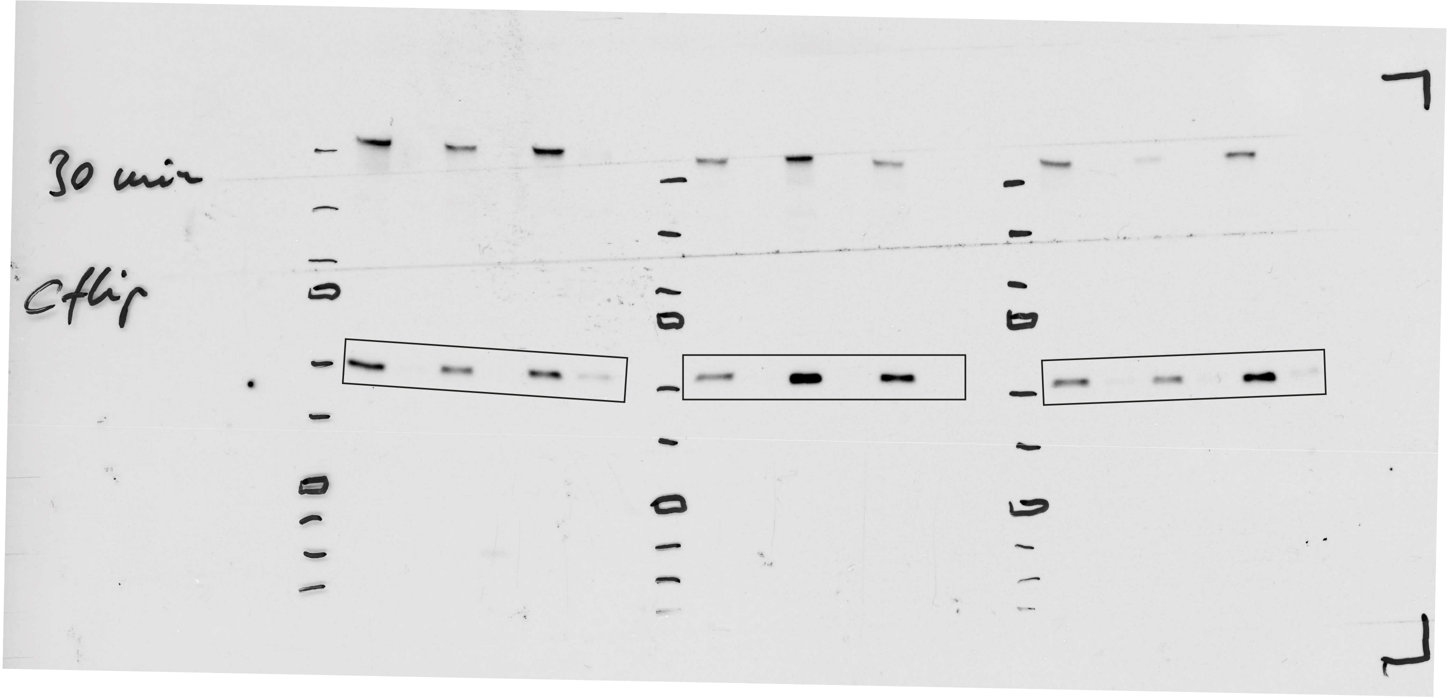

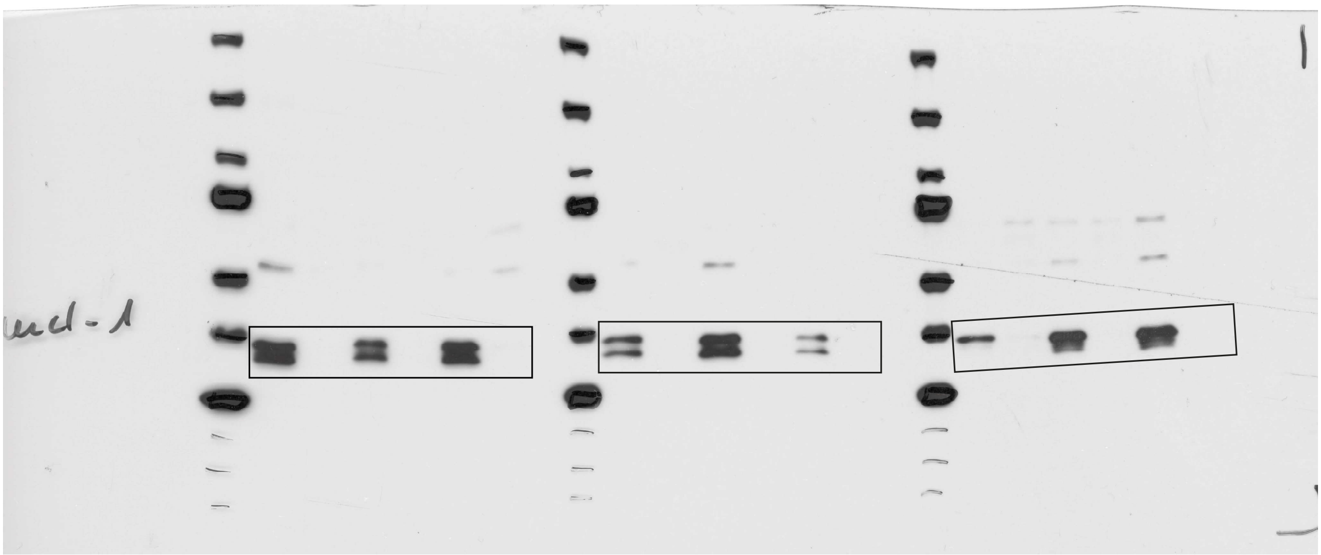

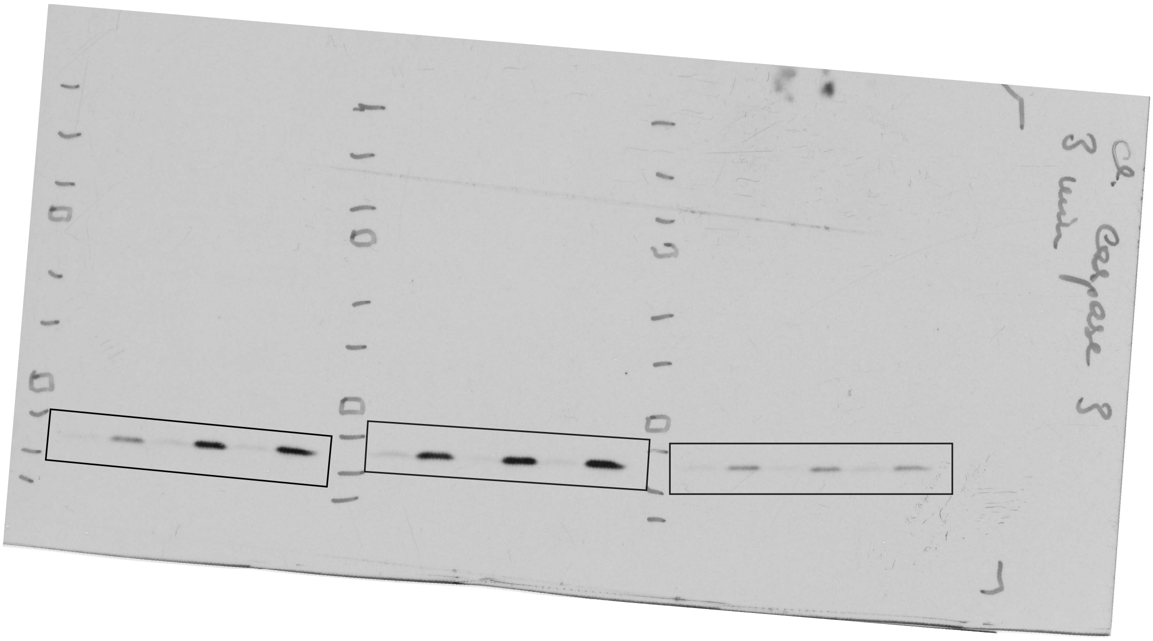

Figure 4G

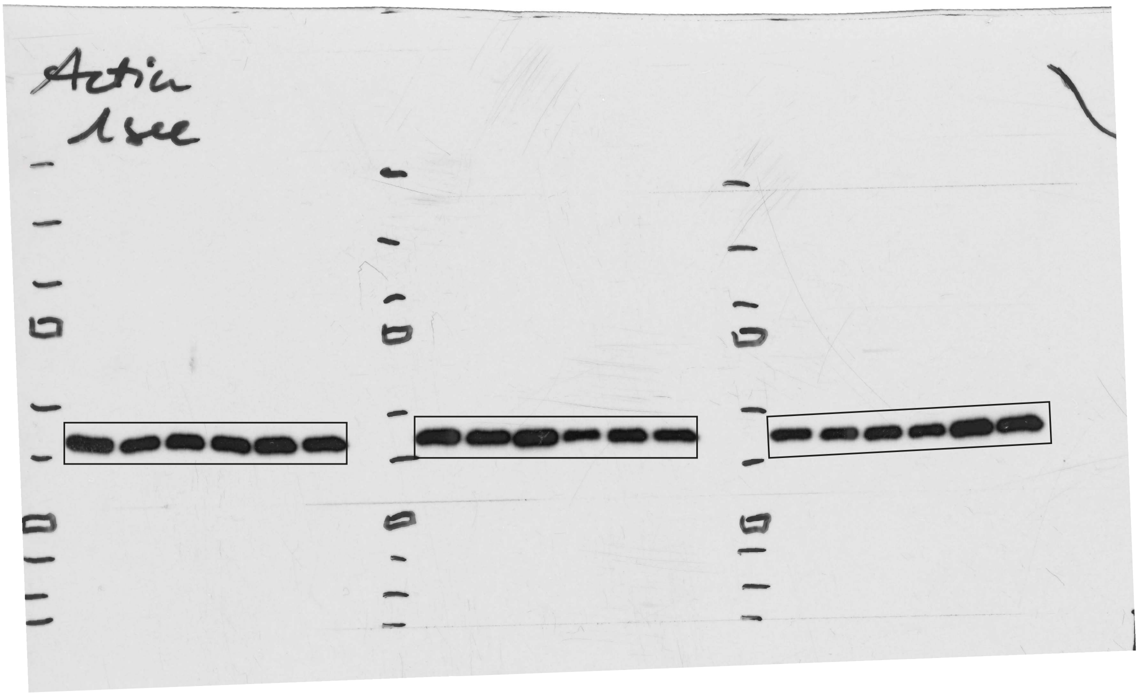

Supplemental figure 1G

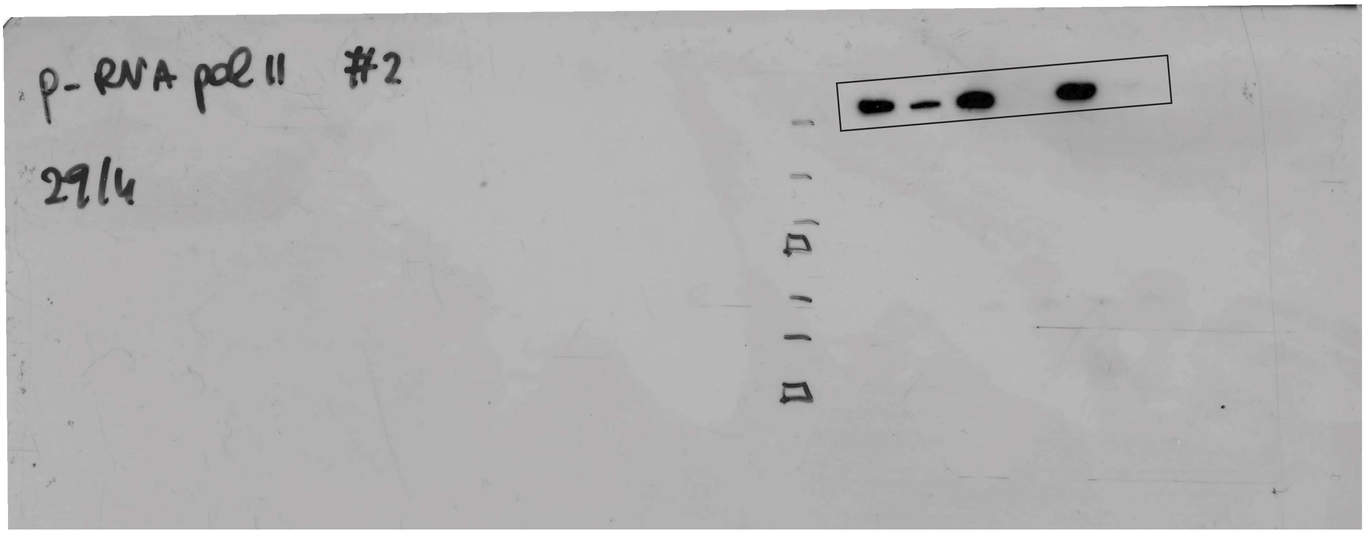

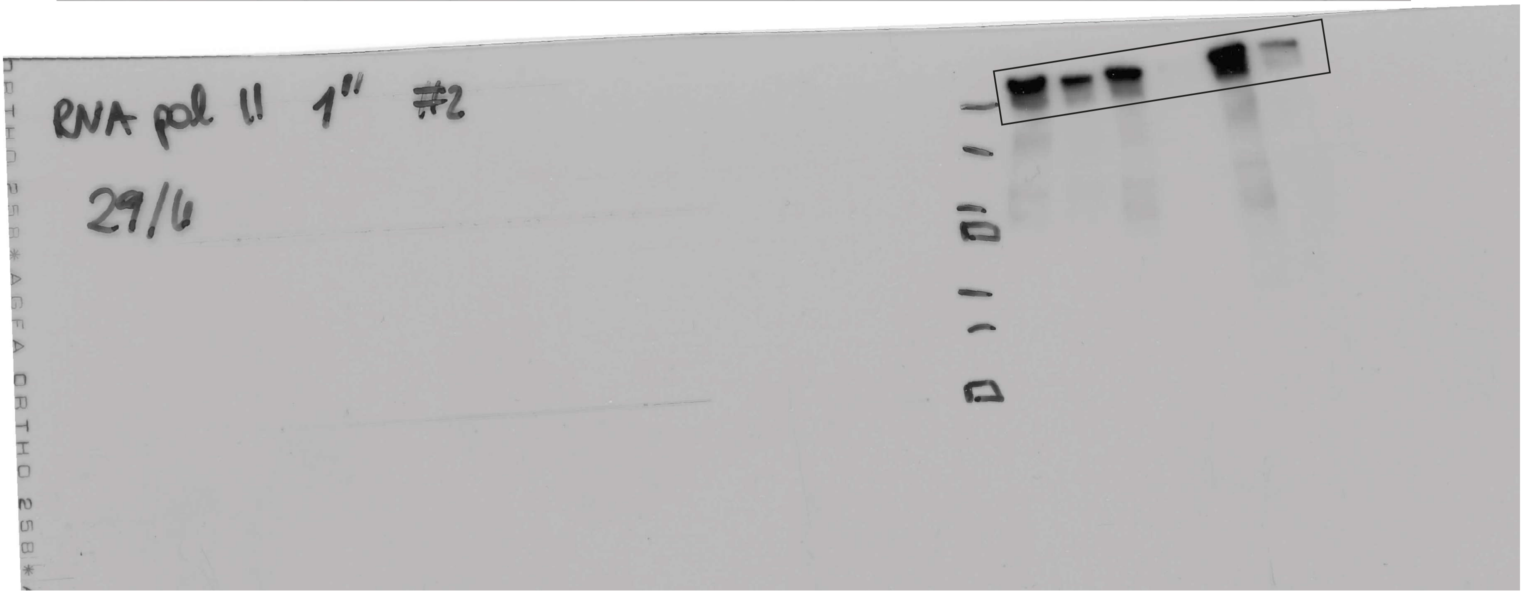

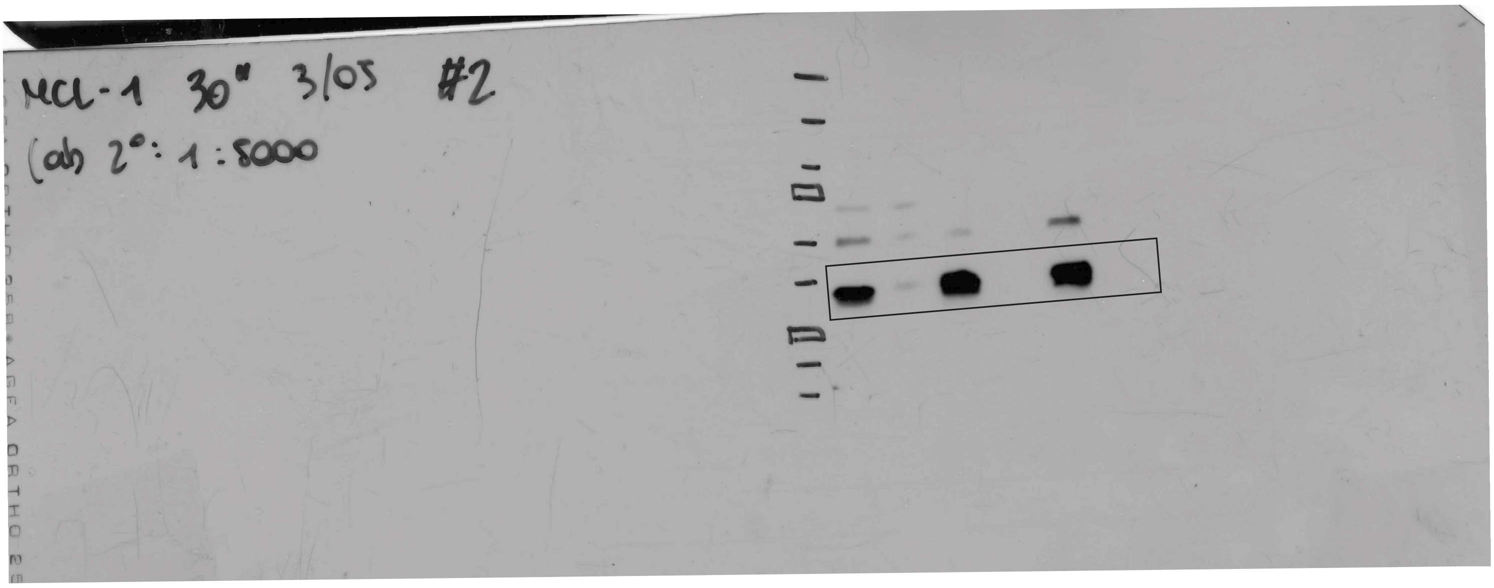

Supplemental figure 1G

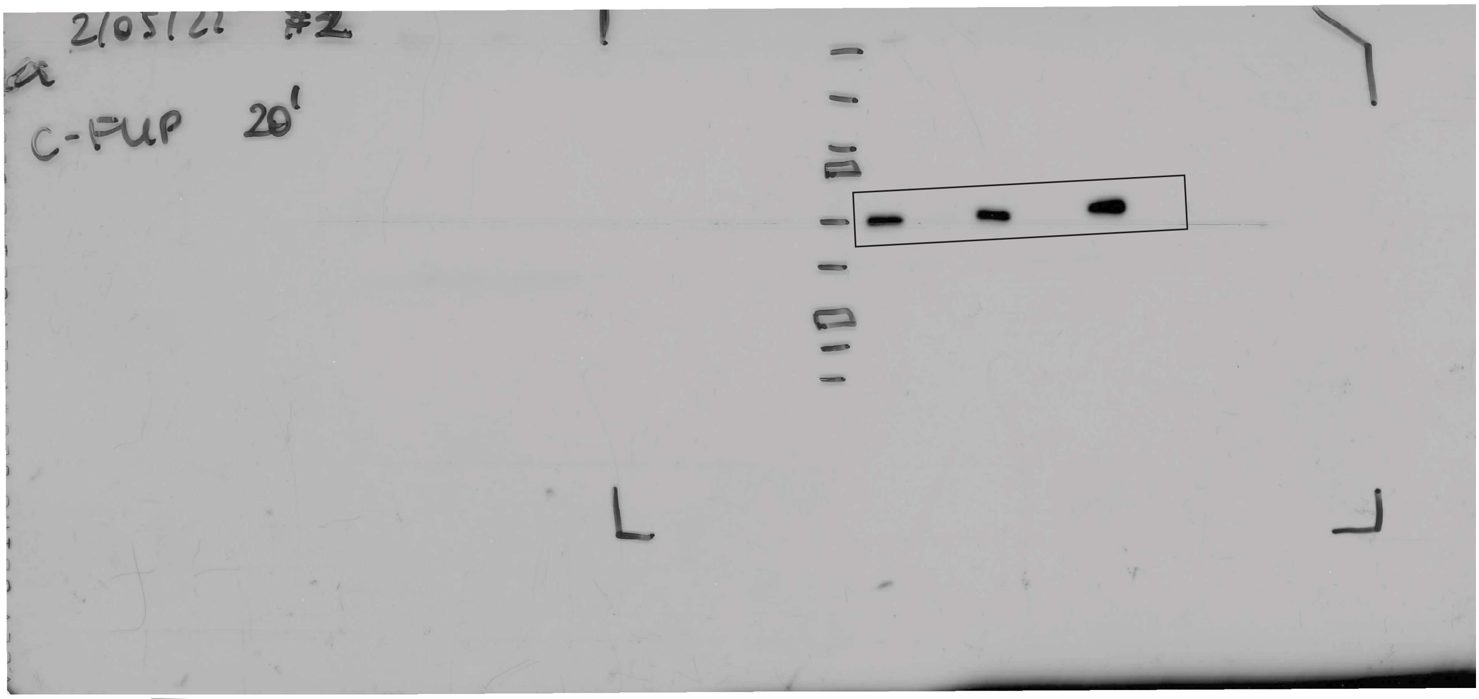

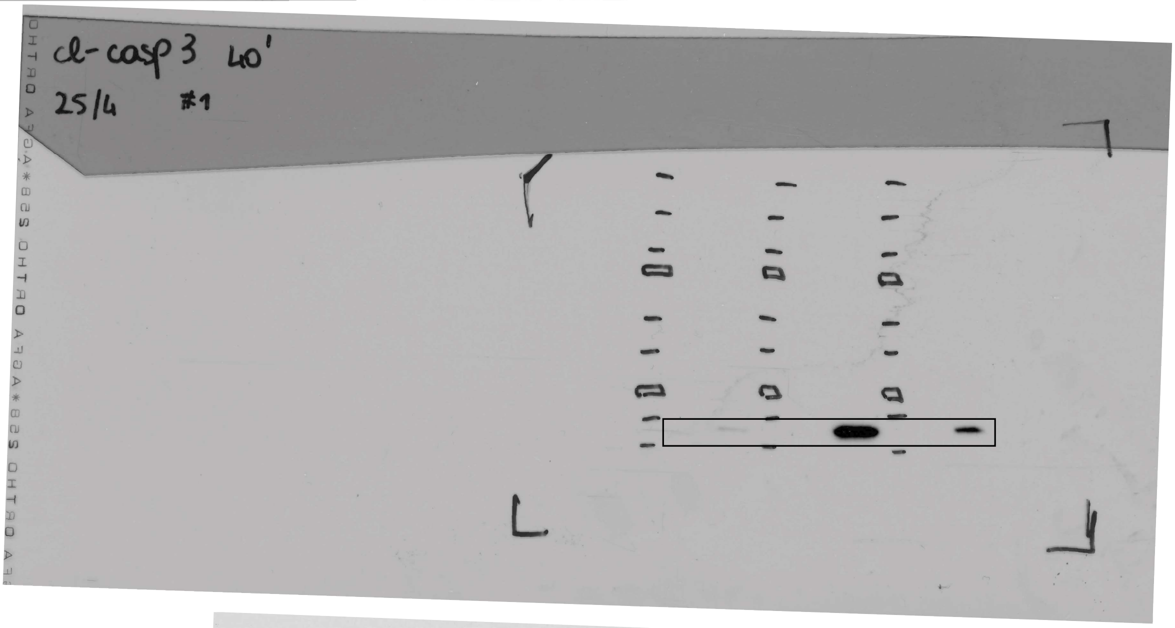

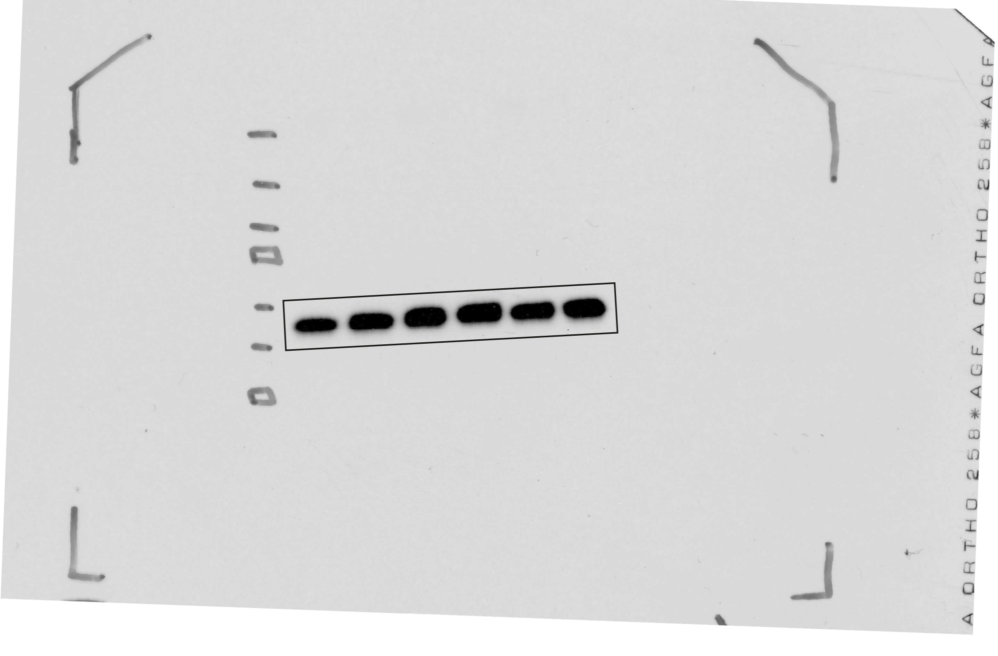

Supplemental figure 2B

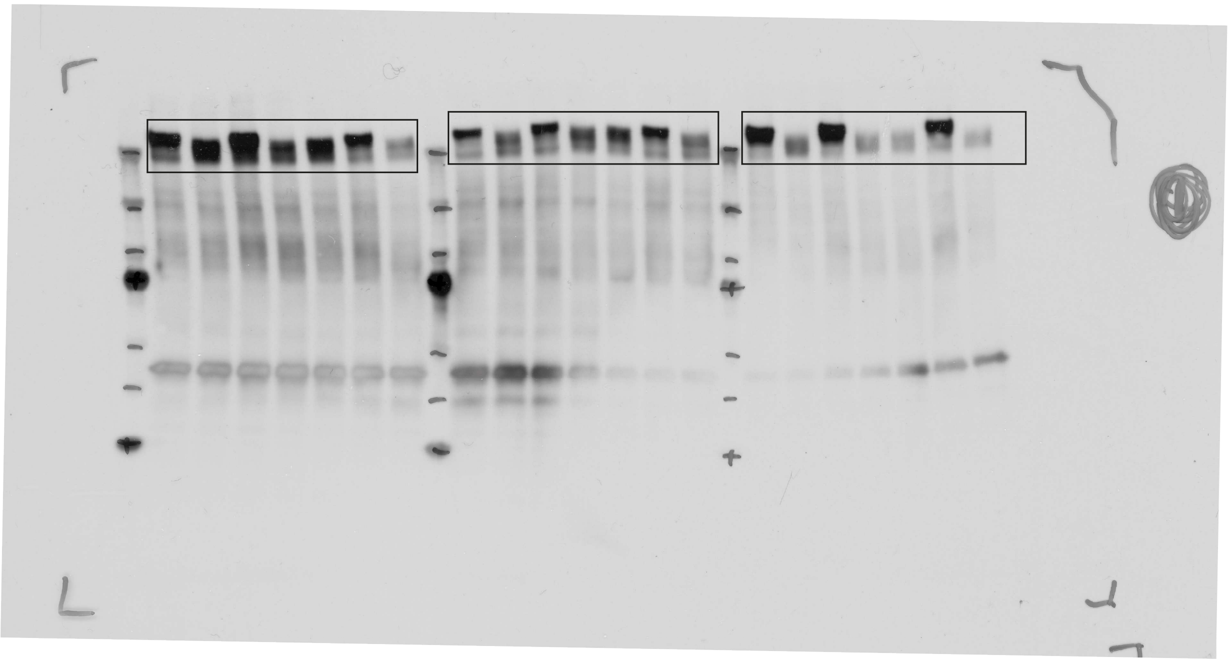

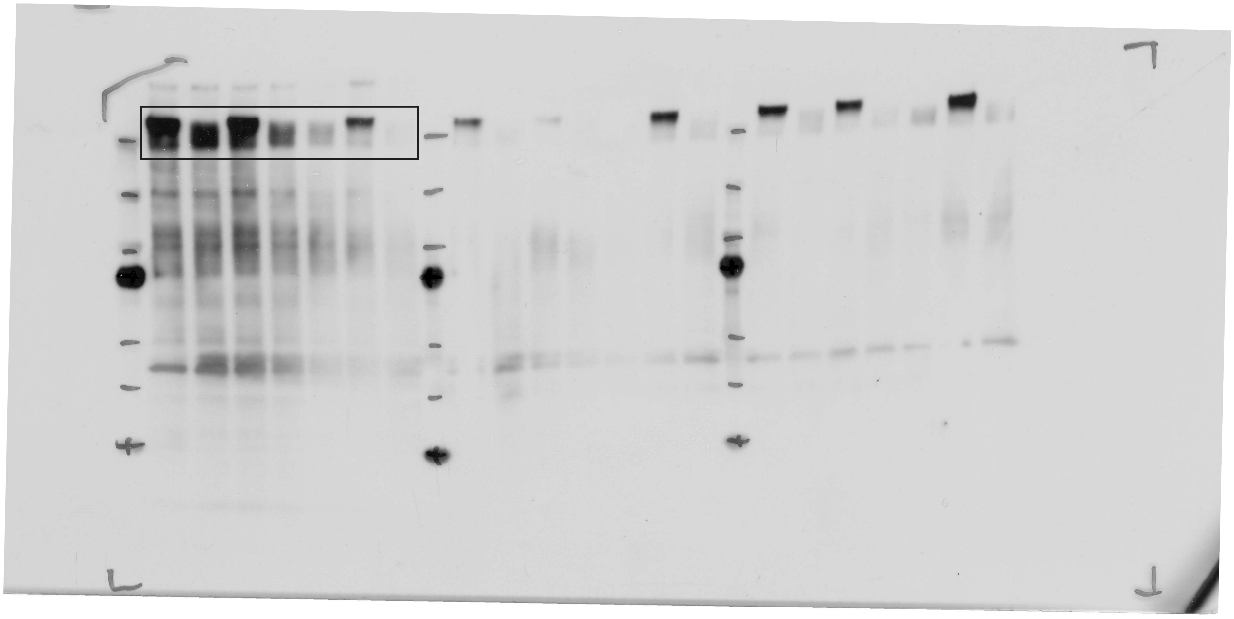

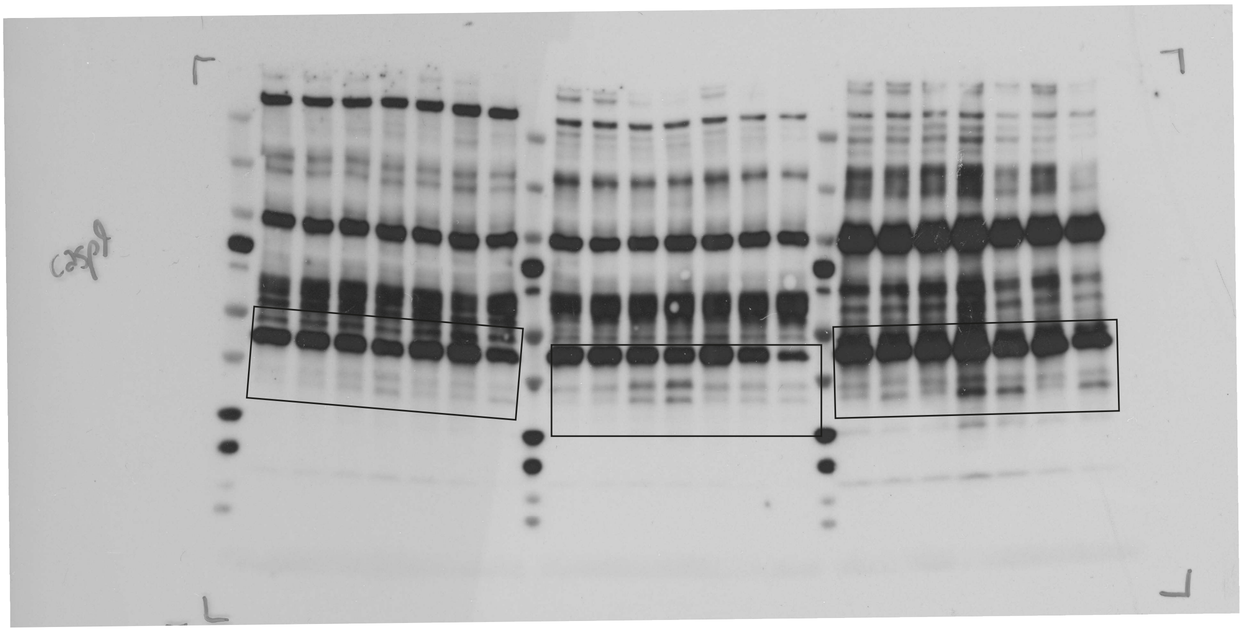

Figure 2C

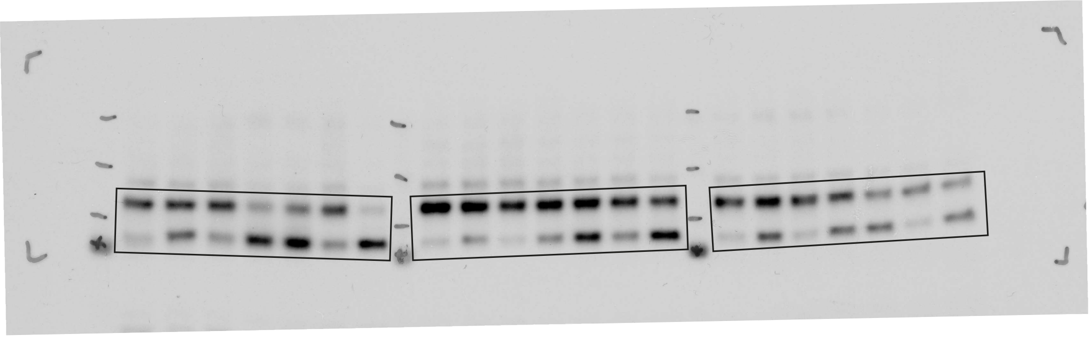

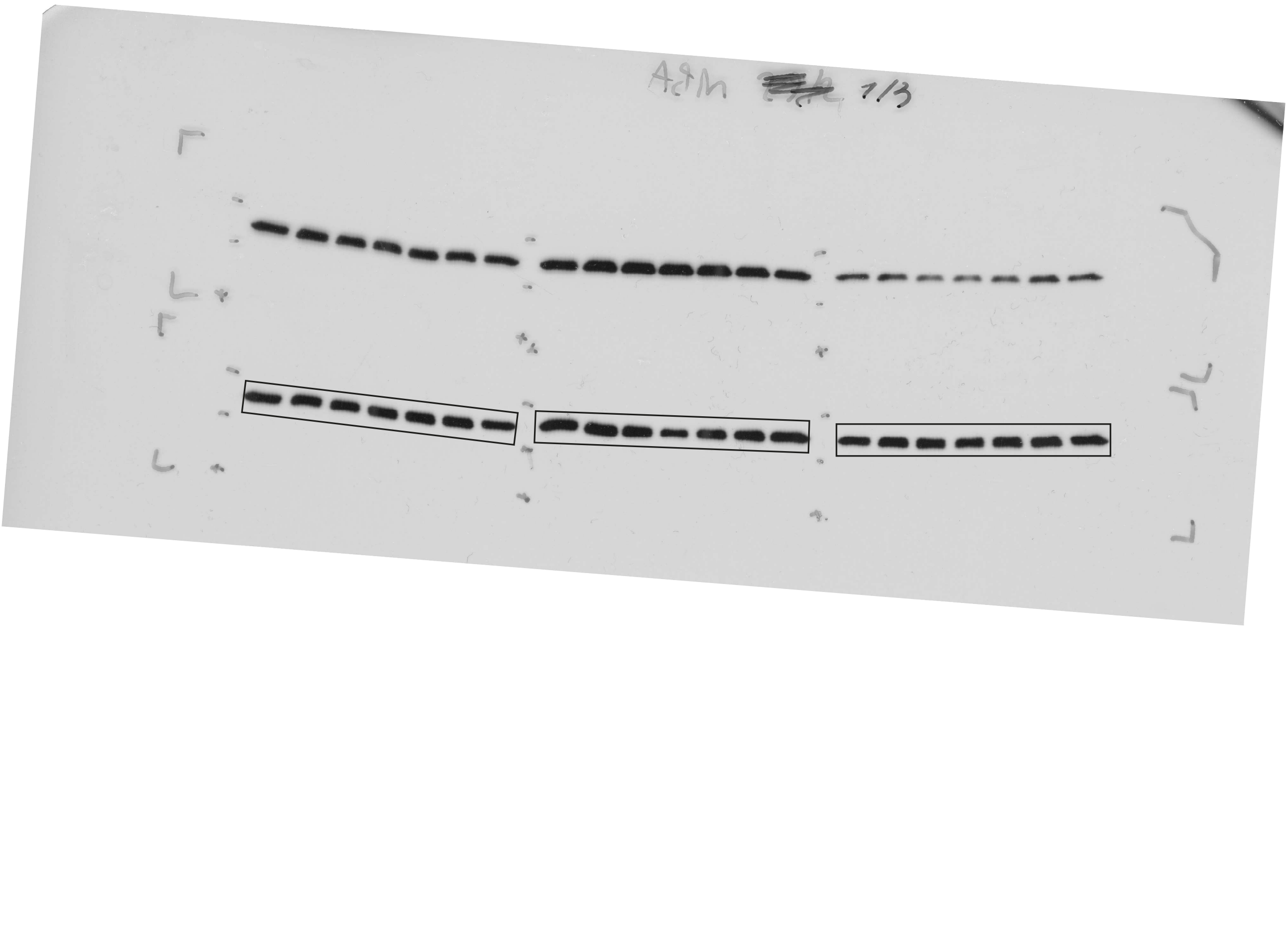

Supplemental figure 2B

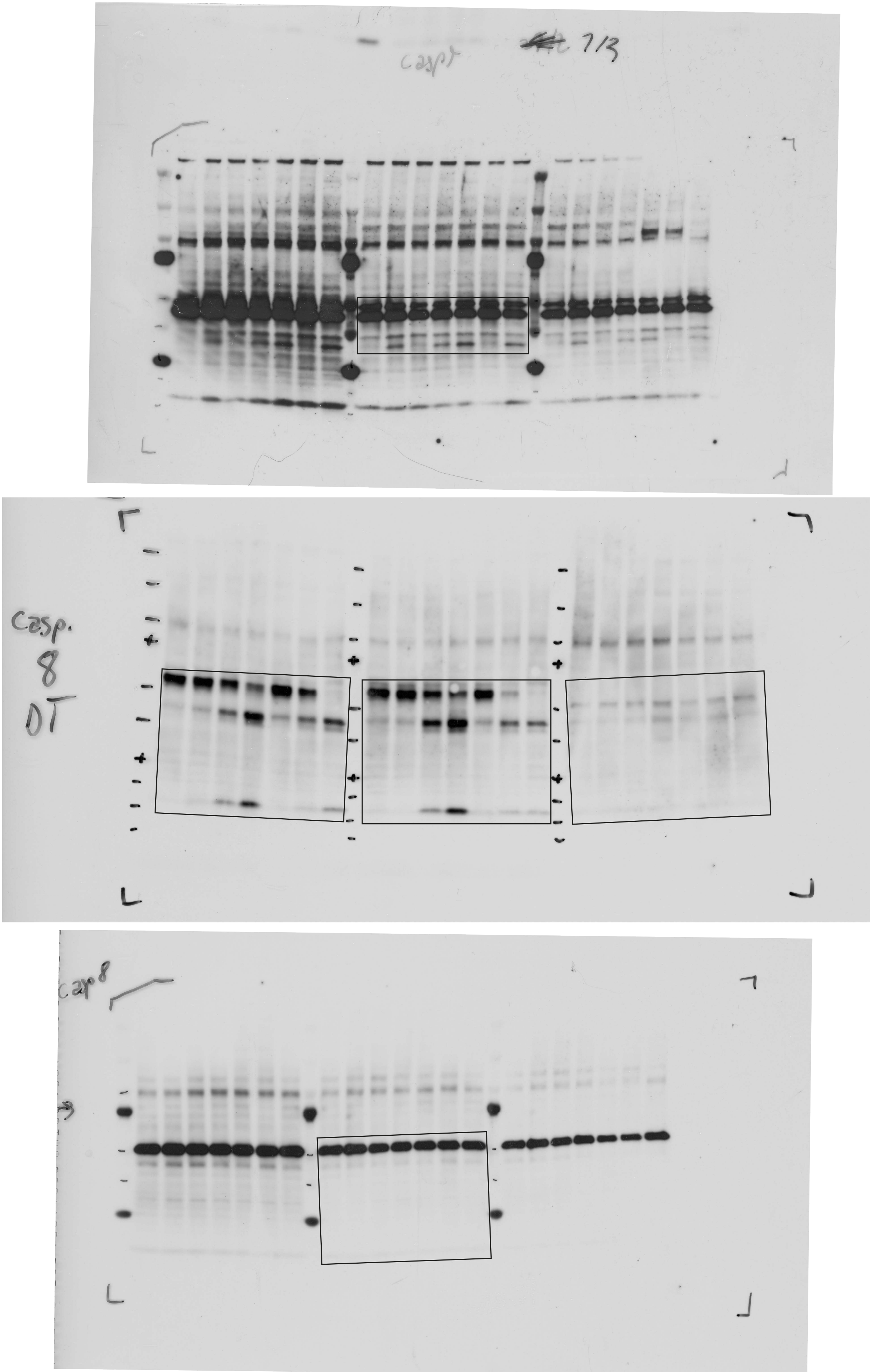

Figure 2C

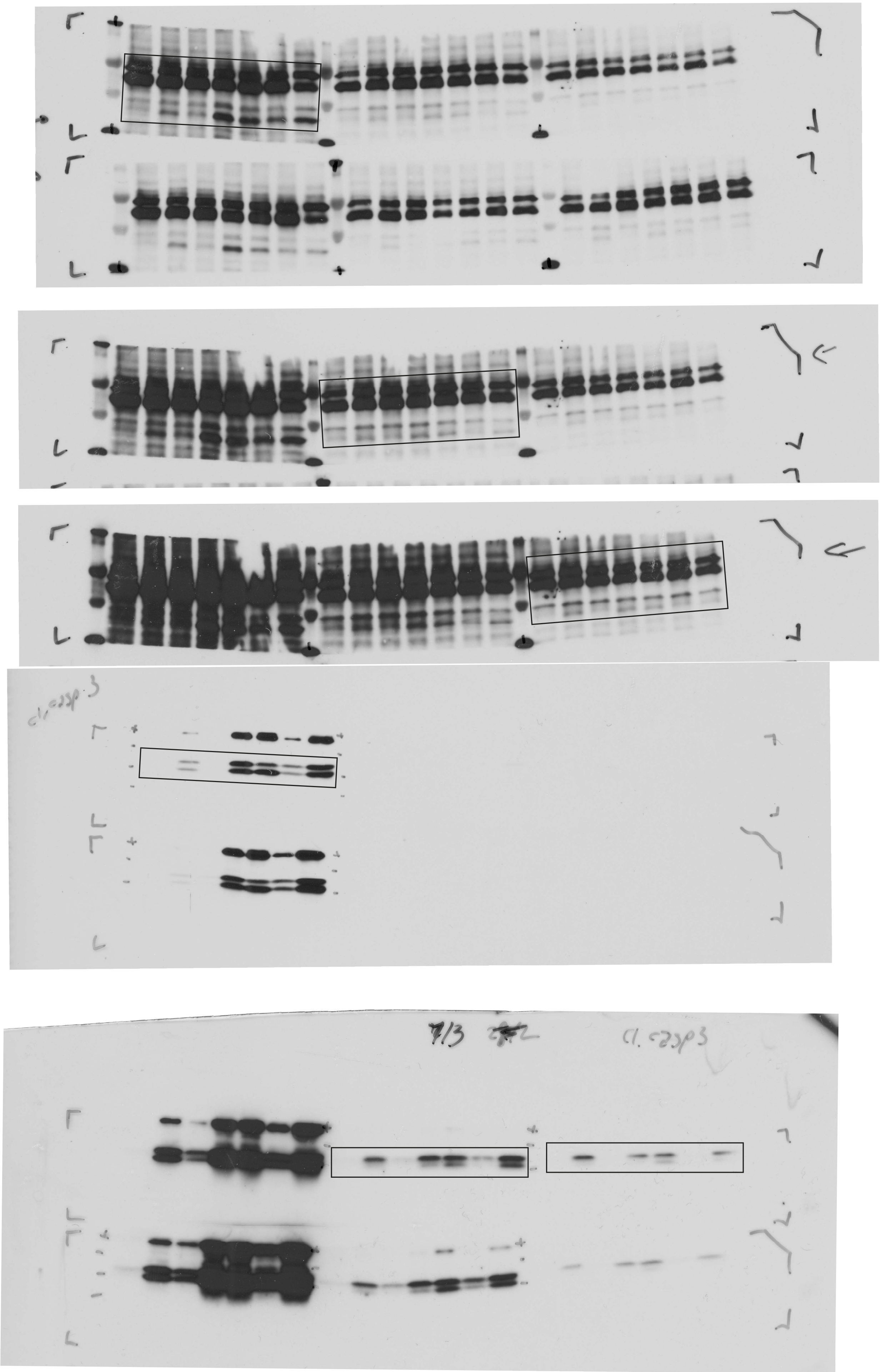

Supplemental figure 2B

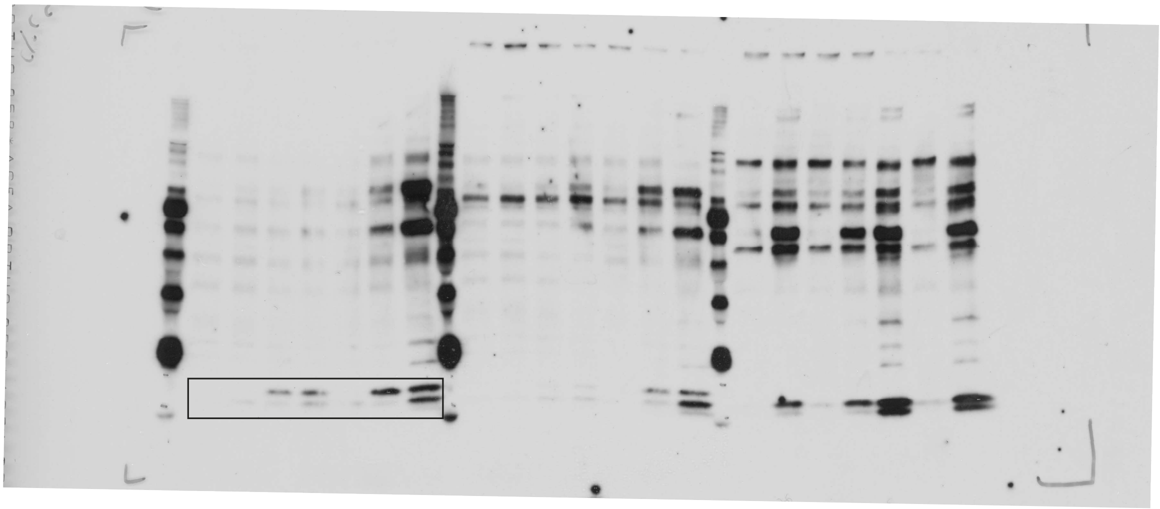

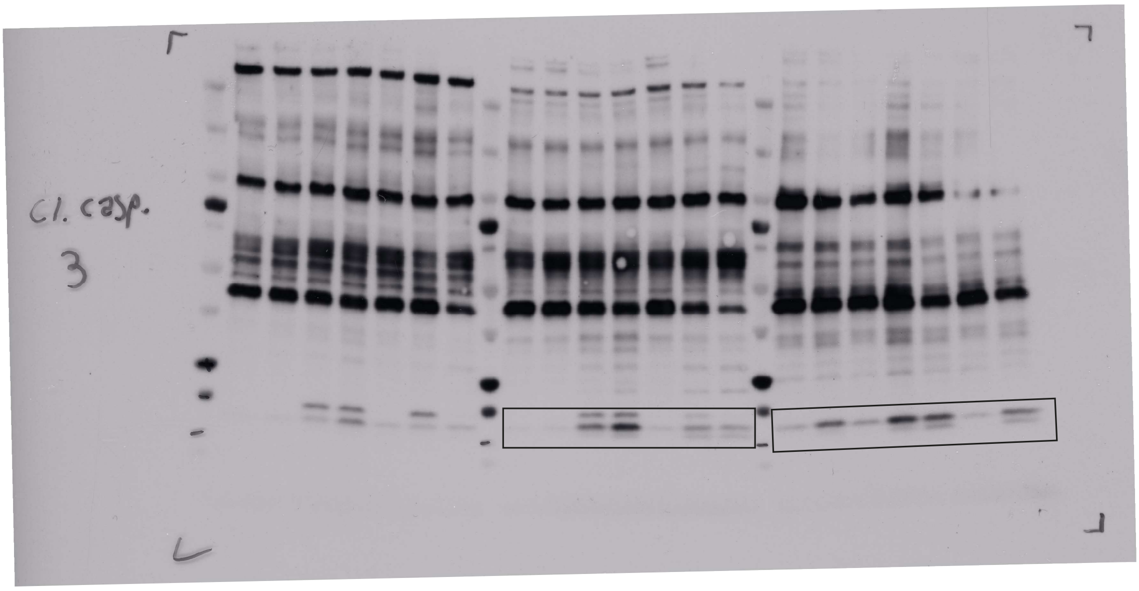

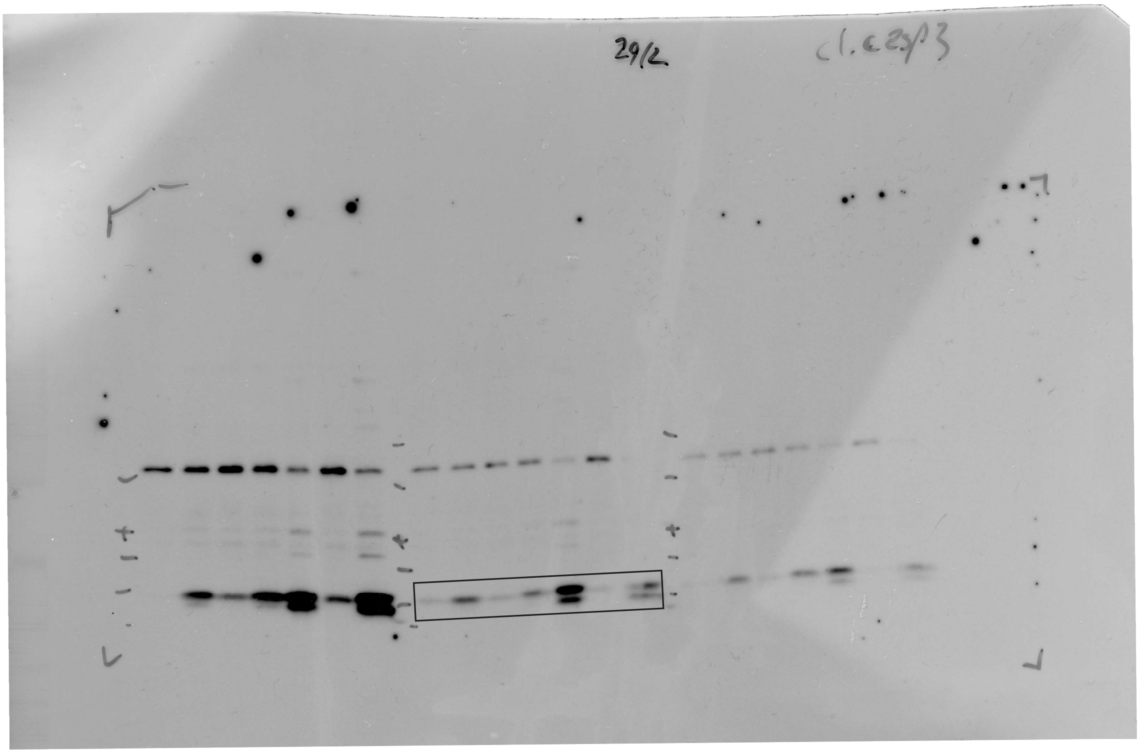

Supplemental figure 2B

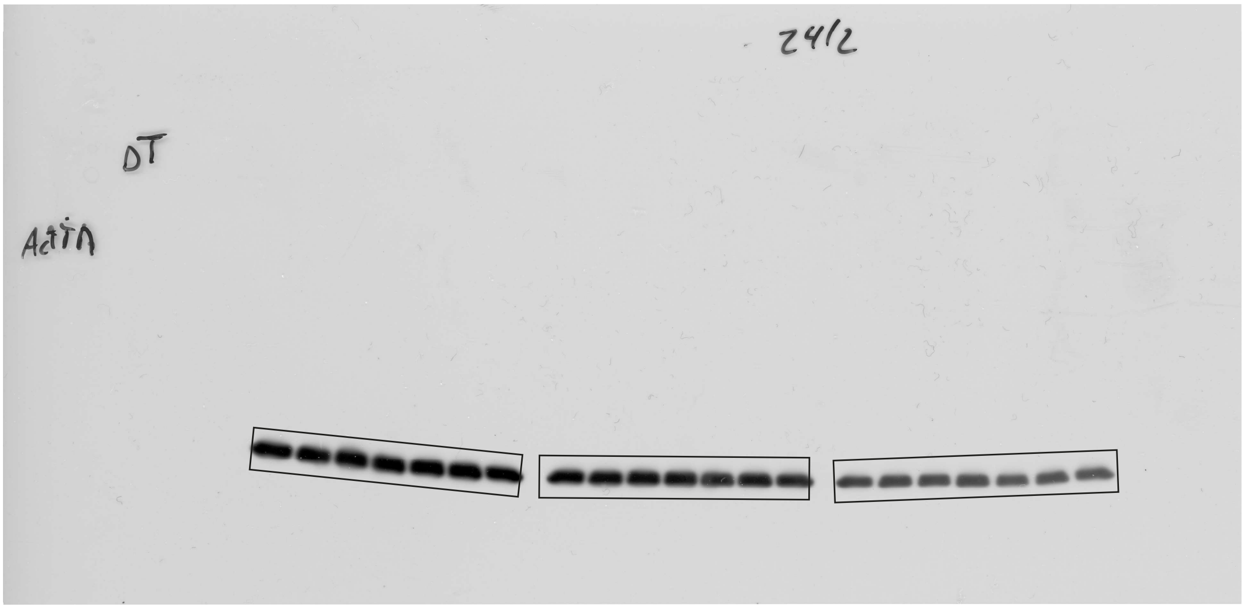

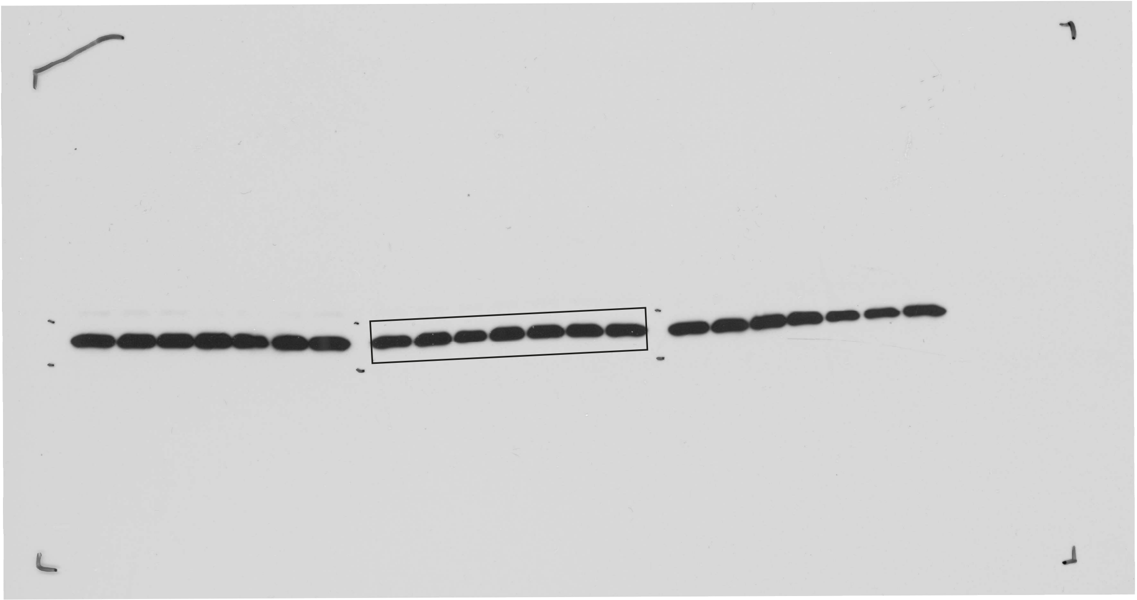

Supplement: Supplementary file 2 — Western Blot full films [file 41419_2024_6724_MOESM2_ESM.pdf]
